# The Gift That Keeps on Giving: Medicaid as a Crucible of Public Goods

**DOI:** 10.1017/amj.2025.10085

**Published:** 2025-12

**Authors:** Robert I. Field

**Affiliations:** 1 Thomas R. Kline School of Law, Drexel University, Pennsylvania, US; 2 Dornsife School of Public Health, Drexel University, Pennsylvania, US

**Keywords:** Medicaid, public goods, health policy, health reform, health security, hospitals

## Abstract

Medicaid has been called the “workhorse” of the American health care system, but one would hardly see that in the tenor of political debates. The Program perennially faces political headwinds that at times build to hurricane force with proposals for dramatic structural changes and spending cuts, most recently the draconian cuts enacted by Congress in 2025. In 2024, Medicaid covered more than seventy million Americans, and another ten million were covered by its companion program, the Children’s Health Insurance Program. As formidable as these numbers are, the Program’s impact runs much deeper, affecting the lives of almost everyone in the United States. It serves as an essential support for the entire health care system and, in doing so, helps to sustain almost every hospital, nursing home, and a range of other providers. This support, in turn, generates population-wide benefits that can be seen as public goods on which everyone relies, whether they realize it or not, that the private sector could not provide. These include peace of mind from knowing there is access to inpatient hospital care, emergency rooms, and long-term care when needed, protection from public health threats, improved health care based on continual innovation, greater social stability, enhanced economic productivity, and reduced health inequities. As devastating as proposals to shrink Medicaid would be for millions of low-income Americans who rely on it for access to health care, these repercussions would cause hardship for almost everyone.

This article explains Medicaid’s role in sustaining the overall health care system, the nature of the public goods it produces in doing so, and the widespread harm that would be caused were these public goods to be diminished. By characterizing public debates in this way, the Program’s supporters could reframe political discourse as a matter of universal self-interest.

## I. Introduction

Medicaid has been called the “workhorse” of the American health care system,^1^ but one would hardly see it that way from the tenor of recurrent political debates over its size and structure. The program has perennially faced political headwinds, recently building to hurricane force. Proposals for structural changes that could substantially shrink Medicaid, such as work requirements, federal funding through block grants, and tighter eligibility reductions, arise with increasing frequency and stridency.^2^ Such proposals are not merely hypothetical. Work requirements and tighter eligibility rules were enacted by Congress in 2025 as part of budget legislation,^3^ yet, as devastating as these proposals are for millions of low-income Americans who rely on Medicaid for essential health care, the repercussions can cause hardship for the entire country in ways not as readily apparent.

A recent example of Medicaid’s impact illustrates the point. There is only one general acute care hospital in a large, highly populated corridor in the inner city of Philadelphia just north of the city’s center.^4^ The area, known as North Philadelphia, is one of the poorest in the city,^5^ already one of the poorest of the ten largest cities in the United States,^6^ and a large proportion of the residents of the area are Black and Latino.^7^ It also has among the lowest ratios of primary care providers to adults in the city.^8^

Hospital access in North Philadelphia shrank significantly in 2019 after the closure of Hahnemann University Hospital, an academic medical center located at the area’s southern edge.^9^ It had been an important source of emergency and advanced high-technology care for residents with limited access to transportation.^10^ It was also the source of thousands of jobs.^11^ Tenet Healthcare, a for-profit national hospital chain, owned it for almost twenty years.^12^ However, located in a poor area, the hospital faced financial challenges as it had a large number of uninsured patients and relied heavily on Medicaid payments for many of the others.^13^ Eventually, Tenet found Medicaid reimbursement insufficient to cover expenses. In 2019, it sold the hospital to a private equity firm, American Academic Health System, which filed for bankruptcy later that year in the face of continuing large losses.^14^

On first blush, it may not seem surprising that a hospital serving a predominantly poor and indigent patient base would be unable to sustain itself financially. However, health care is different in fundamental ways from other industries. It is not only more extensively regulated but also more heavily subsidized.^15^ Every sector of health care rests on a foundation of government regulatory and funding programs that shape its business structure and underlie its financial foundation.^16^ Of the $4.5 trillion that the United States spent on health care in 2022, more than $1,750 trillion, nine percent of the country’s entire gross domestic product, was spent by government programs, most notably Medicaid, to fund health care for those who are financially needy.^17^ Hahnemann Hospital was able to remain financially viable for decades by relying on Medicaid but could not continue to operate when the program’s reimbursement could not keep pace with its needs.^18^

Nevertheless, government spending on Medicaid is frequently questioned in political debates.^19^ Critics routinely ask whether its beneficiaries truly deserve government support and for those who do, how much they should receive, as they during debates over the 2025 budget bill.^20^ Some argue that the system is rewarding many who are undeserving or receiving more than their fair share of taxpayer-funded assistance.^21^

Questions about Medicaid’s value have led to proposals, including some incorporated into the 2025 budget bill, to dramatically shrink it in ways that threaten its scope and effectiveness both directly and indirectly.^22^ Direct threats come from a plan included in the bill to reduce funding by limiting the ability of states to impose taxes on providers to support their share of Medicaid costs, as most of them do.^23^ This would make it more difficult for states to sustain Medicaid funding at current levels.^24^ It is estimated that this could reduce program expenditures by as much as $612 billion over ten years.^25^ Indirect threats come from a plan to reduce enrollment by limiting eligibility in several ways that are also included in the bill.^26^ One would impose work requirements on adults as a condition of receiving benefits along with complex paperwork rules for reporting work status.^27^ Those rules could cause many who are, in fact, working to nevertheless lose eligibility.^28^ It is estimated that this could reduce enrollment by as many as 5.2 million.^29^ Another would impose more frequent eligibility redeterminations for some enrollees, which could cause as many as 700,000 to lose coverage.^30^

This article addresses arguments for shrinking Medicaid by recharacterizing the program as an essential benefit for all of society, not just for those who receive its direct benefits. The article argues that the portrayal of Medicaid as a “safety net” for the “deserving poor” or as an “entitlement” for a fortunate few misses its broader significance as a mainstay of the entire health care system and thereby of the well-being of everyone. In that role, it produces essential benefits that have many of the characteristics of “public goods” — goods that contribute to public welfare but cannot be profitably provided by private markets on their own.^31^ If society is to have them, it must rely on the government to supply them.^32^

Part I of the article provides a summary of the history and structure of Medicaid within the larger context of American health care. Part II explains the economic concept of public goods, and the related concepts of common goods, externalities, and spillover effects. Part III applies these concepts to eight critical societal benefits that Medicaid creates. Part IV explains Medicaid’s major shortcomings while weighing them against these benefits. The conclusion describes a broader conceptual understanding of Medicaid based on this analysis.

## II. Medicaid and its Place in American Health Care

### A. History and Structure

#### 1. Overall Structure

Although private employer-sponsored health insurance and Medicare cover most of the American population, they fail to reach a sizable portion,^33^ almost one-hundred million in total.^34^ These include people who do not work for an organization that offers health benefits, are not the dependent of someone who does, are unable to afford employer-sponsored insurance if it is available, or are too young for Medicare. Without Medicaid, many of them would have no other source of coverage and therefore limited access to health care.

By number of beneficiaries, Medicaid is the second largest source of health insurance in the United States after private, employer-based coverage.^35^ In 2021, it covered 35.1% of Americans under the age of nineteen, 15.4% of adults between nineteen and sixty-four, and 7.4% of adults over the age of sixty-five.^36^ In 2023, its budget equaled more than half the total amount paid by Americans for private insurance.^37^

Medicaid is an example of cooperative federalism, meaning it is structured as a federal-state partnership, and it is available in every state, although its generosity varies considerably between states.^38^ Funding is shared between the federal government and the states based on each state’s average per capita income, and there is no cap on the amount of the federal contribution.^39^ State contributions vary between fifty and twenty-three percent.^40^ Federal law also sets parameters for coverage and eligibility.^41^ The program has generated considerable political debate in recent years over state decisions on whether to accept federal incentives under the Affordable Care Act (ACA) to expand the range of beneficiaries and over cuts contained in the 2025 bill.^42^

#### 2. Medicaid’s Origins

Medicaid grew out of a predecessor program enacted in 1960 to cover hospital expenses for a few categories of the poor. That program, the Kerr-Mills Act,^43^ was the first to provide financial access to health care on a national basis for patients who would otherwise be unable to afford it.^44^ Although coverage under that act was limited, it represented a significant shift in the locus of government health care spending from the states to the federal government.^45^ Kerr-Mills followed the enactment of laws in twenty-eight states and two territories that provided “old-age assistance.”^46^ Social commentators have observed that the path to their enactment was eased by a softening of widely held attitudes that many of the poor were “social deviates or paupers by choice” and therefore undeserving of government help.^47^

The Kerr-Mills Act created the basic framework on which Medicaid was built with a mix of federal and state financing and administration.^48^ This stands in contrast to Medicare, which was structured as a unified national program financed entirely at the federal level.^49^ The participation of states in Medicaid is voluntary, but as of 1982 when Arizona implemented its program, all fifty states and the District of Columbia had chosen to participate.^50^

In its original form, Medicaid covered a limited group of beneficiaries that included aged, blind, and disabled individuals with low incomes, as well as parents of dependent children receiving public assistance.^51^ States were also required to cover single parents and children receiving welfare through the Aid to Families with Dependent Children (AFDC) program.^52^ However, they were free to set their own thresholds for income eligibility, and many set it below fifty percent of the federal poverty level (FPL).^53^ Under this system of categories, childless adults below the age of sixty-five were ineligible for benefits regardless of income, a situation that was changed in most states by the ACA in 2014.^54^

The original Medicaid program mandated that states determine coverage based on one of two tiers.^55^ The minimal tier required coverage for “categorically needy” individuals, defined as those who qualify solely on the basis of income.^56^ The slightly more generous second tier required coverage for “medically needy” individuals, defined as those who would join the ranks of the categorically needy if medical expenses were considered in determining their financial need.^57^ Covered services for all beneficiaries were mandated to include basic medical care, including hospital and physician services, laboratory and radiology services, and nursing home care.^58^ States that covered medically needy beneficiaries had the option to add coverage for additional products and services, such as prescription drugs, which are covered in all states, and dental care, which is covered in many.^59^

#### 3. Coverage Expansion Over Time

Over time, Congress expanded the scope of Medicaid coverage through both additional mandates for participating states and options that states could choose. A prominent example of an early mandate was the Early and Periodic Screening, Diagnosis, and Treatment (EPSDT) program, created by the Social Security Amendments of 1967,^60^ which requires states to cover regular diagnostic screenings and preventive care for children up to age twenty-one.^61^ Another mandate is for coverage of additional essential services for children up to age six that was added in 1981 to combat concerns over rising infant mortality rates.^62^ That expansion also mandated eligibility for pregnant women with incomes up to a state’s income threshold for AFDC.^63^ Other mandatory expansions in 1989 and 1990 increased the income eligibility thresholds.^64^

The most significant expansion occurred in 2014 under the ACA, which was passed in 2010. It offered states an enhanced matching share of ninety percent^65^ for coverage of residents with incomes up to 133% of the FPL who had not previously qualified.^66^ This change both raised the income thresholds in states that had set them lower and added a new category of eligibility for adults aged eighteen to sixty-four with incomes below 133% of the FPL.^67^ The ACA also added a number of new benefit options, including the Basic Health Program, which enabled states to add coverage for individuals with incomes between 133 and 200% of the FPL who do not qualify for other government health care programs.^68^ Such programs are currently in place in Minnesota and New York.^69^

The ACA, as originally drafted, gave states the choice of accepting the new category of coverage or losing eligibility for all federal matching Medicaid funds.^70^ In 2012, the Supreme Court ruled in the case of *National Federation of Independent Businesses v. Sebelius* that a penalty that large constituted unconstitutional coercion of the states, but that implementation of the enhanced matching share as an incentive for voluntarily expanding Medicaid was constitutionally permissible.^71^ The expansion thereby became voluntary for states. Forty states and the District of Columbia had accepted it as of 2024.^72^

#### 4. Growth in Enrollment Over Time

The expansion of benefits and eligibility over time led to a substantial expansion of Medicaid enrollment and cost. During the program’s first fifty-five years, enrollment grew by more than twenty-fold, from four million in 1966 to 84.7 million in 2021.^73^ There were two periods of especially rapid growth, one in 2014 resulting from implementation of the ACA expansion, and one in 2020 with the start of the COVID pandemic and a temporary prohibition of disenrollment.^74^ Spending during those fifty-five years increased from $0.9 billion to $735.4 billion, reaching more than 17.9 percent of all health care expenditures in the country in 2023.^75^ In 2021, Medicaid was the third largest payer for health care services in the United States after private insurance and Medicare.^76^

#### 5. Innovation through Waivers

A distinctive aspect of Medicaid is the various forms of flexibility it gives states to administer their programs in innovative ways. Among the most important is the authority of the Centers for Medicare & Medicaid Services (CMS), the agency that oversees the federal part of the program, to waive some federal operational requirements to enable states to try new approaches.^77^ Most of these waivers are permitted under two sections of the Social Security Act. Section 1115 authorizes CMS to allow experiments through demonstration programs.^78^ An important early example tested alternative ways of paying providers, such as through managed care and prospective payment to hospitals.^79^ Waivers under this provision were also used to expand access to home and community-based services (HCBS) for beneficiaries needing long-term care who would otherwise require institutionalization,^80^ and to increase treatment options for substance use disorders.^81^ Section 1915 of the Social Security Act was added in 1981 to permit CMS to let states vary their Medicaid programs on an ongoing basis.^82^

In improving access to care for these vulnerable populations, waiver programs also support a range of specialized providers that render their care.^83^ These include professionals, such as occupational therapists and physical therapists, organizations that provide outpatient care, and providers of ancillary services, such as transportation and educational support.^84^ This coverage also reduces the uncompensated care burden on hospitals.^85^

#### 6. Children’s Health Insurance Program

As critical as Medicaid is to the health of millions of low-income Americans, another safety net programs provides important additional support for targeted populations. The Children’s Health Insurance Program (CHIP), enacted in 1997, provides federal matching funds for states that cover children in families with incomes slightly above the Medicaid threshold.^86^ Under it, states can add that coverage either as a separate program or as an expansion of Medicaid.^87^ All states have implemented CHIP coverage, with some increasing eligibility to as high as 200% of the FPL.^88^ In April 2025, CHIP covered more than seven million children.^89^

### B. Medicaid’s Importance for Providers

#### 1. Transition From Public Hospitals to Subsidized Coverage

Until the middle of the twentieth century, government support for indigent hospital care was provided primarily through public hospitals, most of which were located in major cities.^90^ However, as the cost of operating these facilities rose in the latter part of the century and Medicare and Medicaid became available to finance care in private hospitals, a growing number of cities moved away from that model, and many public hospitals were closed.^91^ In their place, those cities turned to Medicaid to support care in private facilities,^92^ which were then forced to take up the slack.^93^ As of 2020, only 498 of the 5,230 hospitals in the United States were public.^94^

As more public hospitals around the country closed, the need for government support of private facilities serving their former patients grew. An example is the closure in 1977 of Philadelphia General Hospital, which had been a model of public hospital care for decades.^95^ In its place, indigent patients were sent to several private hospitals, where their care was reimbursed by Medicaid and other government programs. Another example is the closure of Atlanta Medical Center. When plans were announced in 2022 to close it, city and state officials scrambled to find ways to find public funds to relieve the expected strain on a large private hospital system, Grady Health System. The closure left Grady’s flagship hospital as the operator of the region’s only Level 1 trauma center.^96^

As the burden of indigent care has grown for private hospitals, some have incurred large amounts of debt, which has increased their risk of closure.^97^ In fact, debt resulting from indigent care has been identified as the greatest threat to hospital financial survival.^98^ Hospitals’ financial shortfalls are magnified by a failure of Medicaid and other public funding sources to fully cover the cost of care.^99^ The closure of Hahnemann is a notable example, but there are numerous others.^100^ A particularly disruptive one was in Chicago, where Mercy Hospital and Medical Center, a 412-bed facility on the city’s south side that served a predominantly low-income population, closed in 2021.^101^ It was one of three closures of major hospitals in the city that involved facilities in predominantly Black neighborhoods.^102^ Indigent care and insufficient reimbursement have also forced many rural hospitals to close, leaving residents with substantial geographic barriers to accessing care.^103^

#### 2. Support Through Disproportionate Share Payments

To improve the finances of struggling safety net hospitals, defined as those that deliver a significant amount of health care to patients who have no insurance or are on Medicaid,^104^ state Medicaid programs enhance their reimbursement with supplements known as “disproportionate share” (DSH) payments.^105^ Since states are not required to set Medicaid base payment rates at amounts that reflect the actual cost of care, the rates are usually lower than those provided by Medicare and most private insurance plans.^106^ DSH payments are intended to make up some of the shortfall.^107^

DHS payments began in 1981^108^ and constitute a significant share of the amount that many hospitals receive from the program and of program spending.^109^ In 2021, states paid a total of $18.9 billion in DSH payments, with $8.1 billion coming from state funds and $10.8 billion from federal funds.^110^ In 2015, they accounted for forty-nine percent of Medicaid inpatient hospital payments nationally.^111^

However, DHS payments vary considerably between states, even though they are mandatory.^112^ A report by the Government Accountability Office found that in 2017, they represented ninety-seven percent of payments to DSH-eligible hospitals in Maine and only 0.7% in Tennessee.^113^ The report also found that nationally, they covered only fifty-one percent of actual uncompensated care costs.^114^

DSH payments are essential to the ability of many facilities to provide care to lower-income patients.^115^ Moreover, they have been shown to have a significant effect on the financial stability of hospitals that serve those patients.^116^ For many facilities, they are an important source of revenue.^117^ With these supplements, Medicaid reimbursement for some conditions is comparable to or even higher than Medicare rates.^118^ In addition to helping hospitals compensate for shortfalls in Medicaid reimbursement and for the cost of care for patients without any insurance, DSH payments have been instrumental in enabling many hospitals to handle surges in demand, as occurred during the early stages of the COVID pandemic.^119^

#### 3. Support From the ACA Medicaid Expansion

For hospitals, the ACA’s Medicaid expansion has further reduced the number of low-income patients whose care is uncompensated.^120^ As a result, it has been an important source of support for many that serve predominantly low-income populations, as documented by research comparing the financial state of hospitals in jurisdictions that expanded Medicaid and those that rejected it.^121^ An important consequence has been a reduction in the financial disparity between hospitals that treat a disproportionate share of low-income patients and those that do not.^122^ A study that compared uncompensated care costs for hospitals in expansion states and non-expansion states found a decrease after the expansion of between 4.1 and 3.1% in expansion states and no change in non-expansion states.^123^ The hospitals benefiting most were those with the highest proportions of low-income and uninsured patients.^124^

Financial assistance from the Medicaid expansion has been identified as an important factor in saving many hospitals from closure. A study published in 2018 found that hospitals in expansion states were six times less likely to close than those in non-expansion states.^125^ The study did not include states that expanded Medicaid after 2014, raising the possibility that the effect nationally may have become even larger over time.

A study conducted in 2022 by the American Hospital Association found that seventy-four percent of rural hospital closures between 2010 and 2021 occurred in states in which the Medicaid expansion either was not in place or had been in place for less than a year.^126^ During that period, 136 hospitals closed, with the largest number, nineteen, closing in 2020 at the height of the COVID pandemic.^127^ Medicaid, it appears, is especially important to struggling hospitals in times of crisis.^128^

#### 4. Support for Long-Term Care Providers

As important as Medicaid is for hospitals, no sector of health care is more dependent on its coverage than nursing homes and other kinds of long-term care facilities.^129^ In 2015, nursing home stays for 1.4 million people were covered by Medicaid in almost 16,000 facilities.^130^ Medicaid beneficiaries, most of whom were elderly, represented about sixty percent of all nursing home residents, with a total cost to the program of $55 billion.^131^ With an average annual cost of about $82,000 per stay and limited coverage under Medicare, few of them would likely have been able to afford these services without Medicaid.^132^

Support that sustains long-term care facilities also confers indirect benefits on millions of family members and others who serve as caregivers for frail elderly and disabled people.^133^ They have been referred to as “invisible second patients.”^134^ The burden of providing this care generates high rates of psychological morbidity, social isolation, physical ill-health, and financial hardship.^135^ It was estimated to affect 42.1 million family caregivers at any point in 2009 and 61.6 million nationwide over the course of that year, with the value of unpaid labor estimated at approximately $450 billion.^136^ This care burden imposes further costs on caregivers in the form of lost income and on employers in the form of lost productivity of employees whose attention is diverted from their job.^137^ Caregivers for elderly and disabled family members may also have less time to care for their own children, which spreads the burden even further and may have consequences that ripple through subsequent generations.^138^

## III. Public Good and Related Concepts

### A. The Concept of Public Goods

Faith in the efficiency of free and competitive private markets in producing and allocating goods and services is a hallmark of traditional economics.^139^ Markets facilitate free interchange between buyers and sellers, which is at the core of a free-market economy.^140^ However, even the staunchest advocates of free markets acknowledge that they are not efficient at producing and allocating all goods and services.^141^ Markets are not even capable of supplying some of them, including many that are essential to public wellbeing. Economists refer to instances such as this as “market failure.”^142^

For private firms to provide a good or service, two conditions must be met.^143^ First, the seller must be able to limit access to the good or service to those who are willing to pay and to exclude those who are not. Such a product is considered “exclusive.” Second, once the good or service has been sold to one buyer, it must no longer be available for sale to another. Such a product is considered “rival.”^144^ “Public goods” are goods and services that do not meet these conditions.^145^ A paradigm example of a public good is national defense, which is nonexcludable because it protects everyone living in the country regardless of whether they pay, and nonrival because the protection that one person receives does not diminish the protection of others.^146^

Since private markets are not equipped to provide public goods, alternative mechanisms are needed if society is to have them.^147^ These can take the form of private collective action,^148^ however governments have the resources and public interest to be most effective at providing them.^149^ Economists describe three forms that government intervention can take.^150^ First, the government can regulate a private market to make it more amenable to competitive dynamics.^151^ Second, it can subsidize the production or consumption of a needed good or service.^152^ Third, it can directly provide a good or service, as in the provision of health insurance through Medicare and Medicaid.^153^

### B. Related Concepts

#### 1. Common Goods

Some important goods and services that the market does not provide meet the criteria for public goods not because of their intrinsic features but because of external actions. These actions are usually the result of government policy.^154^ Such goods are known as “common goods.”^155^

An example of a common good is public education.^156^ Non-excludability is not an intrinsic feature of it, since tuition could be imposed and students excluded for nonpayment. However, the government makes it nonexclusive by mandating that it be free and available to all children. It is also rival, since the supply of classrooms and teachers is limited, and a seat taken by one student is unavailable for others. Government intervention makes it nonrival, or at least less rival, by building more schools and hiring more teachers. This essential public benefit thereby gains key features of a public good, although it does not fit the idealized concept.^157^

A system of universal health care in those nations that have it can be seen as a common good.^158^ Like education, health care services can be denied to those who do not pay, but under a universal system, they are made nonexclusive by virtue of law. While the elements needed to provide them, such as facilities, drugs, and clinicians, are in finite supply and therefore rival, governments can subsidize their production to reduce their rival nature.

#### 2. Quasi-Public Goods

Some essential goods and services that have some of the attributes of public goods but fail to meet all the criteria may also be characterized as “quasi-public goods.”^159^ Examples include goods that are nonexclusive and nonrival within limits.^160^ Use of a public highway can be restricted by imposing a toll, however it would be infeasible to place tolls on all roads, so most are non-exclusive.^161^ Similarly, one car’s use of a highway does not prevent others from using it, but if enough cars enter the highway, traffic will come to a standstill, making it rival. In response, the government can build more roads or alternative forms of transportation.

#### 3. Externalities

Externalities arise when a transaction affects a party that is external to it.^162^ The effects can be positive or negative. In the case of a positive externality, an outside party receives a windfall, as, for example, when a homeowner installs a smoke detector, which reduces the chance that a fire will spread to neighboring buildings to the benefit of the owners of those buildings. In the case of a negative externality, there is a cost to an outside party, as when a factory emits pollutants that cause health problems for nearby residents and physical damage to their homes.^163^ As with public goods, private markets will not mitigate the problem, since the factory has no financial incentive to reduce emissions.

Externalities, both positive and negative, are also sometimes referred to as “spillover effects.”^164^ These are effects of an activity that “spill over” from that activity to affect others who are not directly involved. In addition to producing consequences for individuals, spillover effects can have broader social consequences.^165^ For example, a positive effect of public education is a more informed and productive population.^166^ A negative effect of a polluting factory is a lower quality of life for an entire region.^167^

## IV. Public Goods Created by Medicaid

Health care services provided to individual patients have the characteristics of private goods.^168^ Clinicians can, and usually do, provide their services only to patients who pay, either with their own funds or those of a third-party payer, making them exclusive. Clinicians also have a limited amount of time to devote to patient care, and in the case of specialists, a limited number of procedures they can perform in a day. Since there is a finite number of clinicians, the supply of these services is exhaustible, making them rival. Similarly, use of a hospital bed is exclusive in that it can be denied to patients who do not pay, and the supply of hospital beds is limited, making them rival.^169^

However, even though the provision of health care to an individual patient is a private good, it creates substantial benefits for the larger society.^170^ Some are significant enough to form foundations of the overall health care system and thereby of many aspects of community and national wellbeing. These benefits are nonexclusive, being available without regard to payment, and non-rival, existing in inexhaustible supply. This leaves the private market ill-equipped to supply them. By expanding the number of people who receive health care, Medicaid makes their creation possible. This Section describes eight of the most important.

### A. Health Security and Health System Sustainability

#### 1. Hospital Viability

In supporting the financial viability of thousands of hospitals, Medicaid promotes health security for the surrounding communities.^171^ This reassurance, in turn, engenders peace of mind for residents in knowing that hospital services are available in time of need even if they never actually receive those services. Beyond this psychic benefit, health security increases the livability and desirability of neighborhoods.^172^ Hospitals also provide preventive services that directly protect community residents’ health, as discussed in Subsection E below.

These benefits are public goods. They are non-exclusive, as they cannot be withheld from those who do not pay, and they are non-rival, as one person’s health security does not diminish the supply available for others. Their production would be substantially reduced were it not for Medicaid’s help in enabling hospitals across the country to remain open.

Inpatient hospital capacity is especially important for health security in times of crisis. This was seen poignantly when the demand for intensive care beds exceeded the supply in many hospitals during the early months of the COVID pandemic.^173^ Other kinds of emergencies, such as natural disasters, can also stress hospital resources.^174^ Such crises are infrequent, but when they occur, the consequences of under-capacity can be dire. With fewer hospitals, there would be less capacity to expand when it is needed.^175^

#### 2. Emergency Services

Few of the services that hospitals provide are more vital to a community than emergency care. On an individual level, it is a private good that can be withheld from those who are not able or willing to pay, either with insurance or their own funds. It is available in limited supply, as there is a finite quantity of beds, clinicians’ time, and supplies. However, as with other aspects of hospital care, the availability of emergency services benefits a much wider population than just the patients who receive them.^176^ The assurance that help is available when urgently needed enhances health security regarding a vital concern and the peace of mind that goes with it. This is an especially important concern for segments of the population with special health care needs, such as parents of young children and the elderly.

The Emergency Treatment and Active Labor Act of 1986 (EMTALA)^177^ mandates that every hospital that receives Medicare reimbursement, a category that includes all but a handful, assess and stabilize all patients who come to their emergency departments regardless of ability to pay.^178^ Although this law does not require treatment beyond assessment and stabilization, these services mean that an urgent threat to a patient’s life or health must be addressed. The law also prohibits delays in providing care that is time-sensitive to inquire about coverage.^179^ These provisions make emergency care nonexclusive, giving it the characteristics of a common good.^180^

EMTALA does not require that care be provided without payment.^181^ A hospital may try to collect reimbursement from a patient’s insurance or from him or her directly after care has been rendered.^182^ However, many patients lack insurance or other financial means of making payment, and they present an obvious financial drain on hospitals.^183^ Medicaid makes instances of nonpayment by patients without financial resources far less frequent than it would otherwise be.^184^ Without it, the burden of caring for nonpaying patients under EMTALA would present a much greater threat to hospitals’ financial stability.^185^

Even with Medicaid, a growing number of hospitals have found the cost of maintaining emergency departments unsustainable and have closed them. A study of closures in nonrural areas found a decline from 2,446 in 1990 to 1,779 in 2009.^186^ It found closures to be especially prevalent among hospitals that had the highest share of Medicaid patients among those within a fifteen-mile radius.^187^ The authors noted that emergency department closures cause much more than just inconvenience for patients.^188^ They decrease access for everyone in the community, even those with private insurance, and can disrupt hospital care in an entire region. In addition to increasing the distance to the nearest facility, they increase patient loads at those hospitals that do maintain emergency departments, which can lead to overcrowding and longer waiting times.^189^ Moreover, patients who come to crowded emergency departments are more likely to leave without being seen, which is associated with higher rates of adverse outcomes.^190^ Other research has found that when hospital closures increase the distance to the nearest hospital, mortality rates from medical emergencies, such as heart attacks and traumatic injuries, increase.^191^

Without Medicaid, closures of emergency department would almost certainly be more frequent. There would be many more nonpaying patients, which would make their continued operation less financially sustainable.^192^ Even those who never need to use emergency services would feel the effects.

#### 3. Financial Viability of Long-Term Care Providers

Millions of people who are unable to care for themselves rely on long-term care from a range of providers. Skilled nursing facilities (SNFs) care for patients who are not sick enough to need the services of an acute-care hospital but are unable to perform basic activities of daily living and need intensive ongoing inpatient care.^193^ Less intensive facilities care for patients who need a lesser level of care but are too frail to live on their own, many of whom are elderly.^194^ Other providers offer outpatient care in community settings and in patients’ homes.^195^ As of 2016, there were 15,600 nursing homes in the United States,^196^ 28,900 other long-term care facilities including assisted living facilities,^197^ 4,600 adult day services centers, and 12,200 home health agencies.^198^ More than 8.3 million people received services from at least one of these kinds of providers.^199^

Inpatient stays in nursing homes can be extremely expensive, with a cost that is beyond the means of most patients.^200^ Only a small percentage of Americans have private long-term care insurance that covers the cost.^201^ Medicare, which is available to almost all Americans age sixty-five and over, covers the cost of up to one-hundred days in a SNF immediately following discharge from an acute-care hospital.^202^ However, it does not cover extended stays in these or in less intensive facilities. Medicaid, in contrast, provides coverage as a mandatory benefit in every state.^203^ The coverage is known as “long-term services and supports” and was used by almost six million Medicaid beneficiaries in 2020.^204^

As a result of this coverage, Medicaid is the primary payer for nursing home care in the United States.^205^ It covers sixty percent of the 1.4 million residents in these facilities.^206^ The cost amounted to almost $55 billion in 2015, representing thirty-five percent of state Medicaid spending.^207^ With this level of support, Medicaid is the financial foundation for much of the industry.^208^

Nursing homes do not serve as mainstays of many communities in the same way as acute-care hospitals. They do not tend to be as large, and they serve a smaller segment of the population.^209^ Moreover, they are subject to frequent complaints concerning the quality of care.^210^ Nevertheless, they are an important source of care not only for patients who are unable to care for themselves but also for family members who would have to find alternative sources of care without them. Moreover, many family caregivers who work outside the home would be unable to continue to do so, placing financial pressure on them,^211^ as well as a potentially severe emotional toll.^212^

The services rendered by long-term care providers are private goods in that they can be, and usually are, denied to those without a source of payment, and they are in limited supply. However, even with constrained availability in some areas, their existence provides reassurance for many frail residents and their families that resources for care exist. The resulting peace of mind is inexhaustible and free of charge.

### B. Mitigating Health Care and Social Inequities

#### 1. Improved Hospital Access in Poorer Communities

While entire communities benefit when everyone has access to health care, the availability of those services is not evenly or equitably distributed.^213^ For example, high-end services such as specialty care are used far more by people with higher than with lower incomes, with the same health status.^214^ This is in part because hospitals, physicians and other providers are often more difficult to find in poorer neighborhoods than in wealthier ones.^215^ Such maldistribution in access to and use of health care can diminish not only the health of those who have difficulty finding a source of care but also the health security that a robust health care infrastructure provides.

The effects of Medicaid in increasing equity in access to health care is demonstrated by statistics cited by sociologist Paul Starr.^216^ In 1964, the year before Medicaid’s enactment, Americans with incomes above the poverty line saw physicians about twenty times more frequently than those with incomes below it.^217^ By 1975, the situation had reversed, with poor patients seeing physicians eighteen percent more often than those who were not poor.^218^ In 1964, white people saw physicians forty-two percent more often than Black people.^219^ By 1973, the difference had shrunk to thirteen percent.^220^ In 1963, patients with incomes below $2,000 a year had half the number of surgical procedures per 100 people as those with incomes above $7,500.^221^ By 1970, the rate for the lower income group was forty percent higher.^222^ Starr attributes the change mostly to Medicaid, noting that poor people who were eligible for Medicaid used health care services that year more often than those who were not eligible.^223^

Similar effects are demonstrated by studies of the effects of the ACA’s Medicaid expansion. A study comparing rates of uninsurance in states that expanded Medicaid and those that did not found that rates of uninsurance were 8.2 lower in expansion states and rates of Medicaid participation were 15.6% higher.^224^ Expansion states also had 3.4% fewer reports of inability to afford follow-up care and 7.9% fewer reports of worries about paying medical bills.^225^ In the first states to expand Medicaid under the ACA, primary care physicians saw an increase of twenty-nine percent in Medicaid visits.^226^ By one estimate, the program’s expansion under the ACA saved the lives of 19,200 adults aged fifty-five to sixty-four in its first four years alone.^227^

Medicaid is especially important in compensating for lower rates of private insurance among Blacks.^228^ Ongoing disparities in employment and income have resulted in more limited access to employer coverage and more difficulty affording private coverage when it is available, making Blacks more likely to turn to Medicaid as a source of coverage.^229^ In 2023, Medicaid covered 38.2% of Blacks, and employer-based insurance covered 52.1%.^230^ The corresponding figures for whites were 19.8% and 73.7%.^231^

Medicaid also offers states considerable flexibility to try innovative approaches to reducing health disparities through waivers. In particular, several states have requested Section 1115 waivers to address specific social determinants of health.^232^ These include programs to devote funds to help with housing,^233^ and to advancing health equity more broadly.^234^ For example, Massachusetts^235^ and Vermont^236^ have requested authority to direct more spending to collecting data on health disparities. In the words of Medicaid scholar Sara Rosenbaum, “There simply is no counterpart to this special legal authority, one that enables Medicaid to be fully transformational extending beyond the traditional roles of insurance.”^237^

States can also leverage contracts with managed care organizations (MCOs) that administer Medicaid benefits to promote health equity.^238^ Federal regulations require that states publicly post quality strategies for MCOs that include reduction of health disparities.^239^ Several states also require MCOs to implement performance improvement projects that address disparities.^240^ Most states require MCOs to screen new beneficiaries for social and behavioral health needs and to make referrals for social services when such needs are identified.^241^

In addition to ameliorating health care disparities based on income, Medicaid has been found to reduce them between rural and urban areas, especially for children.^242^ During the period from 2014 to 2015, forty-five percent of children in rural areas and small towns were enrolled in Medicaid, as opposed to thirty-eight percent in urban areas.^243^ In fourteen states, more than half of the children living in rural areas were enrolled in Medicaid.^244^

Everyone benefits, either directly or indirectly, when health disparities are reduced. It creates a healthier population in which illness is less prevalent, and it lessens animosities that can be caused by the unequal allocation of essential resources. State Medicaid waivers are particularly important in this regard by permitting experimentation that makes the program, in the words of one analysis, “a beacon of innovation and empowerment of local, on-the-ground voices to shape how the program runs, state by state.”^245^

#### 2. Reduction in Overall Poverty

Beyond its effect in reducing disparities in health and health care, Medicaid has been found to have even broader repercussions in reducing overall poverty. One study of the economic effects of Medicaid examined the relationship between income and affordability of essential expenses.^246^ When health care was considered as one of those expenses, Medicaid was shown to reduce the rate of poverty by 2.5% among those younger than age sixty-five, the age of eligibility for Medicare, assuming they would otherwise be uninsured.^247^ In fostering this reduction, it also reduced disparities in rates of poverty according to race, ethnicity, and single parenthood.^248^

A study of hospital closures during the period between 1990 and 2000 found a long-term decrease in real per capita income in the surrounding communities of about $703 in 1990 dollars.^249^ This was estimated to represent a decrease of four percent during the first year after closure.^250^ The long-term decrease was projected to be 1.5%.^251^ As discussed in Section IIA, without Medicaid, such closures would be far more frequent.

A study of the Medicaid expansion in Virginia under the ACA found that in the year following implementation in 2019, newly enrolled beneficiaries reported decreases in concerns about a range of financial needs, including housing, food, regular monthly bills, credit card debt, and loan payments.^252^ The reductions were similar across all demographic subgroups, suggesting that Medicaid serves as a general antipoverty program for all members of a community.^253^ In addition to the economic benefits, reduction of poverty has been shown to mitigate social factors that contribute to poorer health, such as lower socioeconomic status, lesser educational attainment, poorer neighborhood and physical environment, unemployment, and lack of social support networks.^254^

When the rate of poverty declines, individuals who are no longer poor benefit in many ways, as they are more likely to have access to health care resources, stable housing, healthy foods, and safe neighborhoods.^255^ They are less likely to need assistance from income-based government programs, benefiting taxpayers. Children are more likely to be adequately nourished and educated, enhancing the community’s future wellbeing.^256^ Crime is likely to be lower, reducing a source of daily stress.^257^ Conversely, in the absence of these benefits, higher rates of poverty can make it more difficult for businesses to thrive.^258^ These are positive externalities of Medicaid’s enhancement of access to health care, available as public goods for everyone.

### C. Enhancing Economic Productivity and Growth

Beyond its health and societal benefits, Medicaid is an engine for overall economic growth. Most directly, it sends large amounts of funding to a range of private businesses. In 2019, $313.5 billion in Medicaid funds flowed to managed care companies that administer the program.^259^ For providers of health care services, in 2020, $53.2 billion flowed to long-term care facilities,^260^ $86.8 billion to physicians and providers of related clinical services, $220.8 billion to hospitals, and $34.5 billion to drug companies and pharmacies through the purchase of prescription drugs.^261^ As described below, it also enhances the economy in two important indirect ways: helping to maintain the health of the workforce, and supporting job creation by the health care facilities, most notably hospitals, that it supports.

#### 1. Healthier Workforce

People with better access to care are likely to be healthier, a relationship that is reflected in the findings of several research studies.^262^ From an economic perspective, improved health makes those who are working more likely to be productive.^263^ They are less likely to miss work time^264^ and to suffer from chronic conditions that can reduce their productivity.^265^ One study estimated that an employer with 10,000 workers could face almost $3.8 million in productivity loss each year from ill workers.^266^ When children are the beneficiaries of a government program, for example, through a Medicaid waiver or CHIP, their parents are less likely to miss work to care for them.^267^ Coworkers may also benefit from smoother work routines when there are fewer absences.^268^

Healthy workers are also less likely to file health insurance claims. If their coverage is through an employer plan, this may help to reduce premiums.^269^ If the insurance is through Medicare, Medicaid, or another government program, it may reduce government expenditures.

Nevertheless, many employers are unable to afford the cost of offering health insurance to their workers.^270^ To obtain coverage, many of their lower-paid employees turn to Medicaid and subsidized insurance through the ACA.^271^ For these organizations, the availability of these programs provides a crucial resource for maintaining employee health.

#### 2. Hospitals as Job Creators

In addition to enhancing the health of communities, hospitals are a significant contributor to overall economic vitality, in some communities more than any other industry. The United States has about 5,000 community hospitals,^272^ which admit more than 32 million patients a year.^273^ They generate more than $1.8 trillion a year in spending,^274^ which represents about six percent of the country’s gross domestic product of $30 trillion.^275^ In 2023, Medicaid payments to hospitals accounted for nineteen percent of total hospital spending.^276^

Hospitals require large workforces and, as a result, are major employers.^277^ In seventeen states, they are the largest.^278^ In twenty-two of Pennsylvania’s sixty-seven counties, a hospital is the biggest employer.^279^ The Hospital Association of Pennsylvania estimates that hospitals contributed $186.5 billion to that state’s economy in 2023, an increase of sixty-seven percent from 2010, and they provided 627,255 jobs.^280^ During the first year of the COVID pandemic, hospital employment was a stabilizing economic force in many communities, falling 8.2% compared to 14% for the rest of the economy.^281^ Hospitals also help to support ancillary industries, such as construction, real estate, medical equipment, and pharmaceuticals.^282^

Hospital employment is particularly important for the economies of rural communities that do not have other large employers.^283^ Closure of the sole hospital in a region has been found to have a negative effect throughout the immediate area, with one study predicting a four percent decrease in per capita income within the first year after closure.^284^ Within a fifteen-mile radius of a closed hospital, the study found that per capita income decreased by 0.9% and the rate of unemployment increased by 0.3%.^285^ The long-term decrease was estimated to be 1.5%.^286^

### D. Pipeline of Medical Professionals

After new physicians graduate from medical school, they spend several years training in hospitals as residents and fellows.^287^ However, the teaching and supervision involved is expensive to provide.^288^ There are direct costs of salaries for physician teachers and supervisors and indirect costs of lower productivity when time is diverted from clinical care.^289^ Most of this cost is funded by government programs, primarily through supplements to reimbursement under public insurance.^290^ The Medicare program is the largest funder, but Medicaid plays a major role as the second largest.^291^

As of 2022, forty-four states made graduate medical education (GME) payments through Medicaid to help hospitals with the cost of training future physicians.^292^ Most of these funds went to teaching hospitals, but three states also made payments to medical schools, five to community-based providers with training programs, and four to individual physicians.^293^ Payments in twenty-four states covered indirect as well as direct costs.^294^ In twelve states, payments also covered training of nurses and other health care professionals.^295^

The total amount paid nationwide for GME through Medicaid is substantial. In 2018, it was $5.58 billion, a sizeable increase over the $3.78 billion paid in 2009.^296^ In thirty-nine states, funds were also paid for physician training from general revenues, and in sixteen states funds were contributed by local governments.^297^

While Medicare still outspends Medicaid for GME,^298^ state Medicaid programs have the advantage of being able to experiment with different approaches. One example is the use of data monitoring to identify workplace needs. This forms the basis for allocating funding among medical specialties.^299^

Medicaid funding of GME helps not only the budding clinicians who receive training but also the facilities that train them. It provides many safety net hospitals with the funding needed to cover losses from providing care to Medicaid and indigent patients.^300^ While teaching hospitals are not the only recipients of this funding, they are especially reliant on it.^301^ If they were to close, there would be chaos not only for patients and community members but also for their trainees.^302^ The closure of Hahnemann left 571 medical residents and fellows scrambling to find alternate placements.^303^

An ample supply of well-trained physicians and other health care clinicians is a clear benefit for society at large.^304^ It is a private good on an individual level, limited to those who meet certain qualifications. Moreover, only a predetermined number of training slots are available each year.^305^ However, the intangible benefits to society of having enough well-trained clinicians and a pipeline to replenish it helps everyone.

### E. Public Health Protection

Everyone is better off when a community has less illness and disability, which is the goal of public health.^306^ Community members lead healthier and more productive lives, they are more likely to thrive economically, and they experience less fear that disease will strike them and those around them. Reduction of illness and disability in a community is facilitated by several factors, with access to health care prominent among them.^307^ By enhancing access, Medicaid thereby creates a paradigm example of a public good — a better quality of life for everyone regardless of payment and in inexhaustible supply.

Reducing the spread of infectious diseases was one of the major accomplishments of public health during the 20^th^ century.^308^ One of the most effective tools in this regard is facilitating the widespread provision of one health care service in particular — vaccination.^309^ The World Health Organization has estimated that vaccination prevents 3.5 to 5 million deaths annually worldwide.^310^ More than sixty vaccines have been approved for use in the United States, and more are continually added.^311^ Most are administered to children, and laws in almost every state require that they receive a panel of vaccines before entering school.^312^

Vaccination on a wide scale produces an especially important secondary benefit when enough people receive it. Once the proportion of those in a population who are immune to an infectious agent reaches a critical threshold, that agent no longer has enough susceptible hosts to maintain its presence in the population.^313^ Further spread then ceases, and the community achieves a state known as “herd immunity” in which the disease disappears.^314^ For most diseases, the threshold is about ninety percent.^315^ When herd immunity is reached, even those who remain unvaccinated are protected.^316^

On an individual level, the administration of a vaccine is exclusive in that it can be limited to those who pay.^317^ Once a vaccine dose has been administered to one patient, it is not available for others. However, it produces a tremendous positive externality by reducing the risk of disease for everyone with whom the vaccine recipient comes into contact and for many diseases, herd immunity.^318^ This phenomenon was dramatically demonstrated by a community outbreak of measles in Milwaukee in 1990 producing 1,095 cases primarily in areas with the lowest immunization rates.^319^ In making such outbreaks far less likely, vaccination produces a crucial public good.^320^

Public benefits also accrue from public health efforts to prevent and manage chronic diseases. Conditions such as heart disease, high blood pressure, diabetes, obesity, and depression reduce economic productivity^321^ and can make people more susceptible to infectious diseases they might then spread.^322^ However, these conditions are expensive to treat and manage.^323^ Medicaid covers that cost for millions.

The combined effect of all these public health activities has contributed to the production of what may be the most important health-related benefit of all — a dramatic increase in life expectancy. Average life expectancy at birth in the United States rose from 47.3 years in 1900 to 76.5 years in 1997.^324^ Medicaid helps to assure that medical care improves.^325^ It works alongside other longstanding government public health initiatives like sanitation and clean drinking water which have reduced the prevalence of many serious conditions, such as cholera, dysentery, and polio.^326^

The benefits of public health also have a global dimension. These are recognized by several international organizations, including the World Bank, which has described one public health benefit, pandemic preparedness, as a public good that enhances health security for people in every country.^327^ The task of providing this security on a global level falls largely to the World Health Organization, but that institution is perennially underfunded for the magnitude of the challenge, placing much of the burden on efforts of individual countries.^328^ In the United States, a large portion of that burden is met by Medicaid, which plays a key role in pandemic response in two ways. It finances prevention in the form of vaccination and treatment of those who become ill, and it facilitates recordkeeping that public health officials use to track disease spread.^329^ In this regard, it extends the program’s public health benefits globally.

### F. Healthier Children

Research shows that children who are healthier are more likely to grow up to be healthier as adults.^330^ The health of children has been a key objective of Medicaid since its inception.^331^ It is the reason that EPSDT was added as a mandatory benefit in 1967.^332^ Medicaid has grown into the source of coverage for more than forty percent of all births in the United States^333^ and, along with CHIP, for more than forty-five million children.^334^ The same premise lay behind the enactment of CHIP in 1997.^335^

Medicaid coverage of children coincided with substantial improvements in child health for which it was likely a major contributing factor. In the first full decade of Medicaid’s existence (1965-1975), infant mortality declined by thirty-five percent, neonatal mortality by forty-one percent, deaths in early childhood by twenty-four percent, deaths among school-age children by twenty-six percent, and deaths among older adolescents and young adults by twenty-five percent.^336^ During the same period, there was also a tremendous decline in the incidence of several childhood diseases, including measles, mumps, and rubella, which has been attributed largely to increased coverage of vaccinations.^337^ One analysis of Medicaid’s role in promoting the health of children concluded:

Medicaid coverage has provided the foundation on which a comprehensive pediatric health care program is based. Without Medicaid, low-income children would not have full access to well-child visits, immunizations, lead screenings, vision and hearing services, dental care, developmental screening, adolescent counseling services, mental health care, long- term care and treatment for chronic illness. Without Medicaid, low-income females would not have full access to prenatal care and coverage of family planning and other obstetric services that are vital to the health of their newborns.^338^

Research has also identified effects of Medicaid participation in early childhood on social and economic wellbeing in adulthood.^339^ A study of educational attainment found that expanding health insurance coverage for low-income children increases rates of high school and college completion.^340^ A study of occupational success found that children who gained coverage through expansions of Medicaid and CHIP in the 1980s and 1990s paid more in income taxes at age twenty-eight, indicating that they had higher earnings.^341^ The researchers estimated that by the time these childhood beneficiaries reached age sixty, the government would have recouped fifty-six cents in taxes for every dollar spent on coverage.^342^

As with other health care services covered by Medicaid, those provided to children are private goods when considered on an individual basis. Access can be restricted based on payment, and the capacity of health care providers to render them is limited. However, by funding health care for many children whose families would otherwise be unable to pay for it, Medicaid significantly reduces their exclusive nature and lends them characteristics of a common good. This, in turn, helps to extend their effect of creating a healthier adult population as a public good for all of society.

### G. Promoting Health Care Innovation

In its role as a source of support for the health care system within which private providers operate, Medicaid has been a driving force for innovations to address challenges in health care delivery and finance. A notable example is the transition of thousands of disabled, frail, and developmentally disabled patients from institutional care to HCBS, which would not have been possible without the flexibility provided by the waiver provisions of the Medicaid Act discussed in Section I.^343^ Among the more dramatic effects of HCBS is a substantial decrease in unmet health care needs among children with autism spectrum disorder.^344^ Other research has found that HCBS reduce the odds of having unmet medical needs more among Black children than among white children, thereby helping to reduce health disparities.^345^

Beyond innovation in financing, Medicaid has been crucial to innovation in the pharmaceutical industry. The most important source of direct funding for pharmaceutical innovation is the National Institutes of Health (NIH), in its support for biomedical research.^346^ However, Medicaid, along with Medicare, provides important indirect support by financing the use of novel treatments and the training of clinicians who apply them.^347^ This has been particularly important in the development of treatments for cancer.^348^

Medicaid’s coverage of prescription drugs is a major source of program costs, leading Congress to place limits on the amounts that drug companies can charge the program.^349^ Nevertheless, the value of the Medicaid market to those companies, $32 billion in 2018, is considerable.^350^ That year, beneficiaries in Medicare and Medicaid accounted for forty-five percent of spending on prescription drugs in the United States.^351^ By providing this large proportion of the pharmaceutical industry’s revenue, Medicaid supplies critical financial support to make the development of new products financially attractive.

### H. Reducing the Societal Burden of Illness

Perhaps the most fundamental public good of all those produced by Medicaid is a reduction in the burden of illness on society. Without Medicaid and CHIP, many of the tens of millions of Americans covered by those programs would be unable to obtain health insurance, which would make it difficult to receive any health care services.^352^ If they did receive services, they would be responsible for the cost, which could send many into bankruptcy.^353^ Primary care might still be available in public clinics, if any existed in a patient’s area, however wait times can sometimes be substantial.^354^ With the closure of most public hospitals decades ago, there would be few alternative sources of care.

The potential effects of reducing Medicaid participation can be seen in the effect of uninsurance on the millions of Americans who experience it. Most people without insurance lack a reliable source of care, which increases the risk of developing an array of chronic diseases.^355^ For those who have developed one of them, it increases the chance that the disease will be managed poorly.^356^ They are less likely to receive follow-up care, more likely to be hospitalized for avoidable reasons, and, if they are hospitalized, less likely to receive diagnostic and therapeutic services.^357^ Because of consequences such as these, lack of coverage is also associated with shorter life expectancies.^358^ Moreover, measures of health status have consistently been found to be lower for racial and other minority groups that face barriers to accessing care.^359^

By one estimate, poorer health for the uninsured already creates an aggregate national cost of at least $65 billion and possibly as high as $130 billion a year.^360^ Without Medicaid, the cost would almost certainly grow substantially and affect many sectors of the economy. Federal and state governments would face greater demand for whatever public health care services were available. The burden on public health programs would grow to address increased health threats. Many hospitals and clinicians would find it more challenging to care for all their patients with time and resources diverted to caring for a sicker population.^361^ Premiums would likely rise for private insurance to cover the increased reimbursement that hospitals would need to fund their greater uncompensated care load. The benefits of avoiding this burden reach everyone, and they are available in unlimited supply and without regard to payment.

## V. Limits of Medicaid

While Medicaid and other health care safety programs bring the numerous societal benefits discussed in Section III, their limits should also be acknowledged. Two of them are especially significant. First, despite the size of these programs, there are still substantial limits to their reach. Second, in supporting the ability of hospitals and other private providers to render care, they also enable some forms of provider abuse.

### A. Limits to Medicaid’s Reach

Although Medicaid provides financial support that keeps many hospitals afloat, its reimbursement rates, even with DSH supplements, often fail to cover the actual cost of care.^362^ A study of the effects of the ACA Medicaid expansion on hospital finances found that while it led to substantial reductions in expenses for providing uncompensated care, the savings were offset by the gap between Medicaid reimbursement for that care and the cost.^363^ Moreover, states often set Medicaid payment rates for physicians at low levels to control costs.^364^ Nationally, the rates average about two-thirds of those paid by Medicare.^365^ This, along with the administrative burdens of participating in the program, has led many of them to decline to take part.^366^ Federal law requires that payment rates be high enough to ensure that beneficiaries have as much access to health care services as people with private insurance, but neither states nor the federal government has consistently enforced this rule.^367^ As a result, those without access to public health clinics can still encounter difficulty finding a physician whom they can afford to see.^368^

Moreover, while Medicaid has substantially reduced inequalities in access to health care and in overall health, significant gaps based on social factors remain. Notably, access remains more limited for those with lower incomes in most parts of the country, resulting in diminished health status.^369^ Research has also shown that while the ACA Medicaid expansion lowered uninsurance rates overall, it left rates for noncitizens substantially higher than those for citizens.^370^

In terms of provider abuses, although health care providers operate with a mission to improve the wellbeing of patients and the public, self-interested behavior is rampant. Hospitals, both for-profit and nonprofit, are business entities, and as such they face incentives to maximize revenue. These incentives lead many to limit access to care based on financial considerations.^371^ The availability of Medicaid reimbursement has not eliminated this practice.

In particular, it is common for hospitals to restrict or deny nonemergency care for patients who have no source of payment.^372^ For emergency care, assessment and stabilization cannot be denied based on ability to pay under EMTALA, but that law says nothing about nonurgent medical needs.^373^ Moreover, when emergency care is rendered and a patient lacks insurance to cover the cost, both for-profit and nonprofit hospitals routinely send bills and bring lawsuits when they are not paid.^374^ It is not uncommon for patients who face such suits to be forced into bankruptcy.^375^ One large midwestern health system, Allina Health, refused for a time to provide any services to patients who were in debt to it for previous care until negative publicity led it to suspend the practice.^376^ Some hospitals even seek ways to avoid treating Medicaid patients because of its low reimbursement rates.^377^

Aggressive business practices also characterize many nursing homes, especially those with for-profit ownership.^378^ These practices include reducing staffing levels and neglecting basic patient needs.^379^ There have also been numerous instances of billing fraud.^380^ An additional serious quality concern is frequent physical abuse of residents.^381^ Enforcement tends to be lax, and penalties are often delayed or avoided through appeals.^382^

### B. The Balance of Limits and Benefits

Nevertheless, acknowledging these limitations of Medicaid is not to deny that it makes substantial contributions to community and national wellbeing. Because of it, as described in Section II, facilities that are vital to public wellbeing, such as hospitals and nursing homes, enjoy a more stable financial footing, health care is more widely available, disparities in access to care are substantially reduced, and the population is considerably healthier than it would be otherwise. The shortcomings of Medicaid are reason to improve and expand it, not to doubt its value.

## VI. Conclusion

Compared to citizens of many other developed countries, Americans tend to place tremendous emphasis on the values of individualism and autonomy.^383^ These values support the notion that with enough drive and determination, people can “pull themselves up by their bootstraps” to gain whatever advantage they want.^384^ From this perspective, rewards come to those who work hard and take responsibility for themselves, and lack of those attributes often leads to misfortune.^385^

In health care at least, that notion is a gross distortion of reality. The concept of “self-reliance” when it comes to accessing life-saving services is at best a myth and at worst a justification for economically destructive and socially regressive government policies.^386^ No element of American health care could exist in anything like its present size or vitality without a foundation of government support, and everyone who uses it is relying directly or indirectly on a vast web of government programs, with Medicaid at its core.^387^ Even more fundamentally, everyone, regardless of what health care services they use, enjoys the social, psychological and economic benefits that Medicaid creates. These are public goods and common goods that no individual or private entity could create on its own.

In addition to its conceptual value, this framing of Medicaid enables a more powerful pushback against proposals to reduce or eliminate it.^388^ Medicaid is much more than generosity for the “deserving” poor, as important and morally compelling as that generosity may be. It is a pillar of the country’s health care system and thereby of the larger social and economic fabric of our society.

Instead of focusing on ways to shrink Medicaid, policy reforms should focus on ways to enhance it. One important way is by expanding enrollment. A larger program would be a more robust program capable of spreading Medicaid’s society-wide benefits more broadly. For example, in contrast to proposals to require more frequent reenrollment as a barrier to participation, the program could help beneficiaries who remain eligible maintain coverage without interruption. An approach to doing so that has proven effective is to have enrollment remain in force for at least twelve months initially and after each redetermination.^389^ Another approach to increasing participation that has a proven track record is to allow Medicaid officials to use information submitted by applicants for other safety net programs in determining eligibility, a process known as “express-lane eligibility.”^390^ Other ideas that could help Medicaid grow are to enable officials to accept partial applications and to determine eligibility in real time.^391^

As Medicaid kept Hahnemann afloat for several decades to the benefit of its surrounding community, it sustains health care across the country to the benefit of communities everywhere. The ripple effects of maintaining a vibrant health care system in turn enhance everyone’s life in fundamental, if not always visible, ways. We are all beneficiaries of Medicaid, and we all have a stake in supporting its survival and growth.

## Footnotes

1
*See, e.g.*, Phil Galewitz, *Republican Proposals to Cut Medicaid Could be Politically Fraught*, NPR (Feb. 20, 2025, at 10:00 ET), https://www.npr.org/sections/shots-health-news/2025/02/20/nx-s1-5303475/republicans-medicaid-cuts-trump-hospitals [https://perma.cc/T3Z9-SRGK].

2
*See, e.g.*, *id.*

3 Act of July 4, 2025, Pub. L. No. 119-21, §§ 71107, 71119, 139 Stat. 72, 295-96, 306-15.

4
*Interactive Hospital Map*, The Hosp. & Healthsystem Ass’n of Pa., https://www.haponline.org/About-PA-Hospitals/Interactive-Map [https://perma.cc/SJZ5-CQKX] (last visited Aug. 18, 2025). The hospital is Temple University Hospital, which is four miles from the center of Philadelphia. *See id.*

5
*See* Jesse Bunch & John Duchneskie, *Philadelphia’s Wealthiest Neighborhoods Have Median Incomes That Are $100,000 More Than the Poorest Areas*, Phila. Inquirer (Dec. 7, 2023), https://www.inquirer.com/news/philadelphia/philadelphia-neighborhoods-median-income-map-20231207.html#loaded [https://perma.cc/2C4A-EKEL] (including map that shows North Philadelphia and neighboring communities are among the neighborhoods with the lowest median incomes in the city from 2018 to 2022); *see also The Changing Distribution of Poverty in Philadelphia*, Econ. League, https://economyleague.org/providing-insight/leadingindicators/2020/12/16/phlpov19 [https://perma.cc/R6YY-9LW2] (last visited Aug. 18, 2025) ("Northeast Philadelphia, Germantown, Overbrook, Cobbs Creek, Southwest Philadelphia, and the lower sections of South Philadelphia saw significant increases in residential poverty between 2014 and 2019.").

6
*See* Katie Martin, *Philadelphia 2024: The State of the City*, Pew (Apr. 11, 2024), https://www.pew.org/en/research-and-analysis/reports/2024/04/philadelphia-2024 [https://perma.cc/UW4F-S5WR] (table 10 presenting median incomes for major U.S. cities lists Philadelphia at number seven, almost $20,000 below the U.S. average).

7 See Bunch & Duchneskie, *supra* note 5 for a map showing that the composition of North Philadelphia West in 2022 to be sixty-nine percent Black in 2022 and of North Philadelphia East to be thirty-seven percent Latino.

8 Elizabeth J. Brown et al., *Racial Disparities in Geographic Access to Primary Care in Philadelphia*, 35 Health Affs. 1374, 1379 (2016).

9 Maria Cramer, *Philadelphia Hospital to Stay Closed After Owner Requests Nearly $1 Million a Month*, N.Y. Times (Mar. 29, 2020), https://www.nytimes.com/2020/03/27/us/coronavirus-philadelphia-hahnemann-hospital.html#:~:text=hahnemann%2Dhospital.html-,Philadelphia%20Hospital%20to%20Stay%20Closed%20After%20Owner%20Requests%20Nearly%20%241,the%20cost%20was%20too%20steep [https://perma.cc/4JHH-WG3W] (“Hahnemann Hospital, which once served the city’s poorest patients, closed in September 2019.”).

10 Chris Pomorski, *The Death of Hahnemann Hospital*, New Yorker (May 31, 2021), https://www.newyorker.com/magazine/2021/06/07/the-death-of-hahnemann-hospital [https://perma.cc/AT6R-AAZ4] (“Hahnemann served mostly low-income patients, but it has a range of medical subspecialties and was the primary teaching hospital used by Drexel University’s College of Medicine.”).

11 Richard J. Hamilton, *The Hahnemann University Hospital Closure and What Matters: A Department Chair’s Perspective*, 95 Acad. Med. 494, 494 (2020).

12 Pomorski, *supra* note 10.

13
*See* Pomorski, *supra* note 10.

14 Harold Brubaker, *Tenet will Leave Philly, Selling Hahnemann, St. Christopher’s to Paladin*, Phila. Inquirer (Sep. 1, 2017, at 11:59 ET), https://www.inquirer.com/philly/business/tenet-leaves-philly-selling-hahnemann-st-christophers-to-paladin-20170901.html [https://perma.cc/4CDG-ME6B] (“In the year ended June 30, St. Christopher’s, Hahnemann, and related operations had $790 million of operating revenue and an adjusted operating loss of $15 million … .”); Pomorski, *supra* note 10.

15
*See* Robert I. Field, Mother of Invention: How the Government Created ‘Free-Market’ Health Care 2 (2014) ("The government funds, directs, and nurtures American health care on a fundamental level. Its role is so pervasive and of such longstanding importance that it can be credited with creating health care as we know it … .”).

16
*Id.* at 24 ("Every core element of the system was fashioned and shaped in one way or another by the government … .”).

17 Off. of the Actuary, *National Health Expenditures 2022 Highlights*, Ctrs. for Medicare & Medicaid Servs. (Dec. 13, 2023), https://www.cms.gov/newsroom/fact-sheets/national-health-expenditures-2022-highlights [https://perma.cc/789L-832V].

18 Nina Feldman, *Many Fear Hahnemann’s Story Will Send a Message: Buying a Failing Hospital Pays*, WHYY (July 31, 2019), https://whyy.org/articles/many-fear-hahnemanns-story-will-send-a-message-buying-a-failing-hospital-pays/#:~:text=Here%20are%20some%20reasons%20why%20Hahnemann%20may,and%20often%20on%20the%20verge%20of%20bankruptcy [https://perma.cc/Y6VT-RW8J] (“One of the reasons Hahnemann failed financially is because two-thirds of its patients were on Medicaid or Medicare — publicly funded insurance plans that reimburse hospitals for care at lower rates than private insurance does."). In an official announcement of its closing, Hahnemann Hospital stated, “Hahnemann University Hospital has been experiencing severe financial difficulties. Despite our best efforts to find solutions, none were found. The hospital cannot continue to lose millions of dollars each month and remain in business.” *For Patients*, Hahnemann Univ. Hosp., https://www.hahnemannhospital.com/Closure%20FAQs%20for%20the%20Community.html [https://perma.cc/8MXP-8G2K] (last visited Aug. 19, 2025).

19 Adam Millsap, *Medicaid Spending is Taking Over State Budgets*, Forbes (Jan. 23, 2020, at 08:32 ET), https://www.forbes.com/sites/adammillsap/2020/01/23/medicaid-spending-is-taking-over-state-budgets/ [https://perma.cc/DVQ7-S67H].

20 Daniel Lanford & Jill Quadagno, *Identifying the Undeserving Poor: The Effect of Racial, Ethnic, and Anti-Immigrant Sentiment on State Medicaid Eligibility*, 63 Socio. Q. 1, 15 (2021). This study examined the relationship between Medicaid eligibility criteria by state with survey data on racial antipathy and found that states with higher scores of racial animus toward Blacks, Hispanics, and illegal immigrants employed the most stringent criteria for Medicaid eligibility, especially for low-income non-parents. *Id.* During debates over the 2025 budget bill, proponents of adding work requirements to Medicaid claimed that they would serve to “encourage employment, foster self-sufficiency, and reduce overall healthcare spending.” April Noel, *The Detrimental Ripple: Unpacking the Negative Impacts of New Medicaid Work Requirements*, Ctr. for Telehealth and eHealth L. (June 6, 2025), https://www.ctel.org/breakingnews/the-detrimental-ripple-unpacking-the-negative-impacts-of-new-medicaid-work-requirements-1#:~:text=Proponents%20of%20these%20policies%20frequently%20assert%20that,Healthcare%2C%202025;%20Robert%20Wood%20Johnson%20Foundatio [https://perma.cc/JSS2-6HG6].

21 Lanford & Quadagno, *supra* note 20.

22

Katie Keith , *Senate Republicans Unveil Budget Reconciliation Package: Unpacking the Coverage Provisions.* Health Affs.: Forefront (June 18, 2025), 10.1377/forefront.20250618.501213 . These proposals are contained in legislation under consideration in Congress at the time of this writing. *Id.*

23 Alice Burns et al., *5 Key Facts About Medicaid and Provider Taxes.*, Kaiser Fam. Found. (Mar. 26, 2025), https://www.kff.org/medicaid/issue-brief/5-key-facts-about-medicaid-and-provider-taxes/ [https://perma.cc/6R5K-GDS4].

24
*Id.*

25
*Id.*

26 Keith, *supra* note 22.

27
*See* Sara Rosenbaum , *A ‘Shift in Kind’: A Medicaid Work Requirement Would Radically Change Health Policy.* Health Affs.: Forefront (June 16, 2025), 10.1377/forefront.20250612.628094 . In the words of one observer work requirements alone would “fundamentally change the nature of the Medicaid program itself*.”* Keith, *supra* note 22. In words of another, “Medicaid work requirements would represent an enormous shift in US health policy. A work mandate would shake the program’s very ability to operate as a foundational part of the health care systems that dozens of states which have elected to participate in the expansion have sought to build.” Rosenbaum, *supra.* States that decline to impose the requirements would face a substantial loss of funding. Rosenbaum, *supra.*

28

Leighton Ku et al., *How National Medicaid Work Requirements Would Lead to Large-Scale Job Losses, Harm State Economies, and Strain Budgets*, The Commonwealth Fund (May 1, 2025), 10.26099/6tcv-fh75 .

29 Michael Karpman, Jennifer M. Haley & Genevieve M. Kenney, *Expanding Federal Work Requirements for Medicaid Expansion Coverage to Age 64 Would Increase Coverage Losses*, Urban Inst. (Apr. 2025), https://www.urban.org/sites/default/files/2025-04/Expanding%20Federal%20Work%20Requirements%20for%20Medicaid%20Expansion%20Coverage%20to%20Age%2064%20Would%20Increase%20Coverage%20Losses%5B61%5D.pdf [https://perma.cc/FH87-4EG8].

30 Edwin Park, Geo. Univ. Health Pol’y Inst. Ctr. for Child. & Fams., Medicaid and CHIP Cuts in the House Reconciliation Bill Explained 3 (2025), https://ccf.georgetown.edu/wp-content/uploads/2025/06/Medicaid-and-CHIP-Cuts-6-13.pdf [https://perma.cc/6SQM-3C8S].

31 Inge Kaul & Ronald U. Mendoza, Providing Global Public Goods: Managing Globalization 78, 80 (Inge Kaul et al. eds., 2003).

32
*Id.* at 80, 90.

33 Katherine Keisler-Starkey & Lisa N. Bunch, U.S. Census Bureau, Health Insurance Coverage in the United States: 2021 3 fig. 1 (Sep. 2022), https://www.census.gov/content/dam/Census/library/publications/2022/demo/p60-278.pdf [https://perma.cc/24EJ-5NFS]. In 2021, private insurance and Medicaid covered 84.4 percent of the United States population, and 8.3 percent had no health insurance at all. *Id.*

34
*Marketplace Enrollment, 2014-2022*, Kaiser Fam. Found., https://www.kff.org/health-reform/state-indicator/marketplace-enrollment/ [https://perma.cc/388Q-EFPF] (last visited Nov. 1, 2022) (noting that 14.5 million individuals were enrolled in ACA Marketplaces as of 2022); Ctrs. for Medicare & Medicaid Servs., July 2022 Medicaid & CHIP Enrollment Trends Snapshot 3, https://www.medicaid.gov/medicaid/national-medicaid-chip-program-information/downloads/july-2022-medicaid-chip-enrollment-trend-snapshot.pdf [https://perma.cc/3DGM-D9GV] (last visited Aug. 1, 2024) (stating that 89 million individuals were enrolled in Medicaid and CHIP as of July 2022).

35 Keisler-Starkey & Bunch, U.S. Census Bureau, *supra* note 33, at 20 tbl. A-1.

36 Keisler-Starkey & Bunch, U.S. Census Bureau, *supra* note 33, at 27 fig. B-4. (showing that most beneficiaries aged 65 and above also receive benefits under Medicare).

37
*NHE Fact Sheet*, Ctrs. for Medicare & Medicaid Servs. (June 24, 2025), https://www.cms.gov/Research-Statistics-Data-and-Systems/Statistics-Trends-and-Reports/NationalHealthExpendData/NHE-Fact-Sheet [https://perma.cc/8R34-RKSM] (reporting that Medicaid spending was $871.7 billion and private health insurance spending was $1,464.6 billion in 2023).

38 Robert Stevens & Rosemary Stevens, Welfare Medicine in America: A Case Study of Medicaid 47 (1974) (explaining that Title 19 of the Social Security Act liberalized and extended federal grants to states for the indigent and medically needy, establishing the Medicaid program); *see* MaryBeth Musumeci & Katherine Young, *State Variation in Medicaid Per Enrollee Spending for Seniors and People with Disabilities* fig. 4, Kaiser Fam. Found. (May 1, 2017), https://www.kff.org/medicaid/issue-brief/state-variation-in-medicaid-per-enrollee-spending-for-seniors-and-people-with-disabilities/ [https://perma.cc/UQ5V-K9P5] (noting that in 2011, per capita Medicaid spending for nonelderly adults with disabilities ranged from $15,000 to $19,000 in twenty-five states and could double to between $25,000 and $38,000 in states such as Wyoming, New York, North Dakota, and Connecticut).

39 Elizabeth Williams et al., *Medicaid Financing: The Basics*, Kaiser Fam. Found. (Jan. 25, 2025), https://www.kff.org/medicaid/issue-brief/medicaid-financing-the-basics/ [https://perma.cc/6PUD-EECB].

40 Dep’t of Health & Hum. Servs., *Federal Financial Participation in State Assistance Expenditures; Federal Matching Shares for Medicaid, the Children’s Health Insurance Program, and Aid to Needy Aged, Blind, or Disabled Persons for October 1, 2020 Through September 30, 2022*, 85 Fed. Reg. 76586, 76588 tbl. 1 (Nov. 30, 2020).

41 Burns et al., *supra* note 23.

42 Patient Protection and Affordable Care Act of 2010, Pub. L. No. 111–148, 124 Stat. 119, 121; *see* Adam Searing, *Medicaid Expansion Debate: Wyoming, Mississippi and Missouri*, Geo. Univ. Health Pol’y Inst. (June 2, 2021), https://ccf.georgetown.edu/2021/06/02/medicaid-expansion-debate-wyoming-mississippi-and-missouri/ [https://perma.cc/S75T-RSM8]. Concerning political debate over Medicaid cuts contained in the 2025 budget bill, see Nathaniel Weixel, *GOP Medicaid Debate Intensifies as Republicans Search for Cuts*, The Hill (Mar. 27, 2025), https://thehill.com/policy/healthcare/5268561-house-republicans-medicaid-cuts/, stating “House Republicans are under the gun to get specific on how they will offset President Trump’s domestic policy agenda, and they will soon need to decide if they will touch the political stove by trying to scale back Medicaid benefits.”

43 Social Security Amendments of 1960, Pub. L. No. 86-778, 74 Stat. 924.

44
*See* Stevens & Stevens, *supra* note 38, at 28-31 (discussing Kerr-Mills, a means-tested program that provided federal funds to states covering health care services for aged, blind, and disabled patients, and introduced the concept of federal standard-setting for health care services).

45
*See* Stevens & Stevens, *supra* note 38, at 30 (explaining that Kerr-Mills did not increase aid to elders but instead shifted the burden of providing aid from a hodgepodge of charity, municipal, and state programs to the federal government). Prior to the Kerr-Mills Act, the federal safety net had primarily taken the form of pensions through Social Security, enacted in 1935. *See* Nicole Huberfeld, *Federalizing Medicaid*, 14 U. Pa. J. Const. L. 431, 441-43 (2011).

46 Stevens & Stevens, *supra* note 38, at 32-33.

47
*See* Huberfeld, *supra* note 45 at 440-45.

48 Social Security Amendments of 1965, Pub. L. No. 89-97, § 121, 79 Stat. 286, 343.

49 Julia Paradise et al., *Medicaid at 50*, Kaiser Fam. Found. (May 6, 2015), https://www.kff.org/medicaid/report/medicaid-at-50/ [https://perma.cc/5KGP-SLA4].

50
*Id.*

51
*Id.*

52
*Id.*

53
*Id.*

54
*Non-disabled Adults*, MACPAC (Jan. 8, 2021), https://www.macpac.gov/subtopic/nondisabled-adults/ [https://perma.cc/HPZ4-ZZHN]; *Medicaid Expansion to the New Adult Group*, MACPAC (Mar. 30, 2023), https://www.macpac.gov/subtopic/medicaid-expansion/ [https://perma.cc/A7DG-VFAQ].

55
*Medicaid 101*: *Eligibility*, MACPAC, https://www.macpac.gov/medicaid-101/eligibility/ [https://perma.cc/PFP6-CHMZ] (last visited Oct. 14, 2025) (“In addition to covering these mandatory categorically needy individuals under Medicaid, states could choose to cover optional groups of medically needy individuals — those who fell within one of the federal assistance categories (aged, blind, disabled, and families with dependent children) but whose higher incomes made them ineligible for cash assistance and whose medical expenses would be deducted when determining countable income for eligibility purposes.”). Programs for the categorically needy carried over from Kerr-Mills to the new Social Security Act of 1935, while the act was also structured to protect the working population from unexpected income loss. Stevens & Stevens, *supra* note 38, at 7.

56 Stevens & Stevens, *supra* note 38, at 61-62.

57 Stevens & Stevens, *supra* note 38, at 61-62.

58 Stevens & Stevens, *supra* note 38, at 61-62.

59 Julia Paradise, *Medicaid Moving Forward*, Kaiser Fam. Found. (Mar. 9, 2015), https://www.kff.org/health-reform/issue-brief/medicaid-moving-forward/ [https://perma.cc/7TEZ-L2HA].

60 Social Security Amendments of 1967, No. 90-248, § 302, 81 Stat. 821, 929.

61
*EPSDT in Medicaid*, MACPAC (Jan. 11, 2021), https://www.macpac.gov/subtopic/epsdt-in-medicaid/ [https://perma.cc/X98G-KCC3] (explaining that all children under age twenty-one enrolled in Medicaid through the categorically needy pathway are entitled to the Early and Periodic Screening, Diagnostic, and Treatment (EPSDT) benefit, which requires states to provide access to any Medicaid-coverable service in any amount that is medically necessary, regardless of whether the service is covered in the state plan, and noting that just under sixty percent of children who should have received at least one initial or periodic screening received one; also describing state responsibility for informing families, ensuring appropriate screenings, and delivering needed treatments).

62 Paradise et al., *supra* note 49.

63 Paradise et al., *supra* note 49.

64 Paradise et al., *supra* note 49.

65
*See* Michael E. Chernew , *The Economics of Medicaid Expansion*, Health Affs.: Forefront (Mar. 21, 2016), 10.1377/forefront.20160321.054035 . The federal share of the cost of expanding Medicaid when it took effect in 2014 was one-hundred percent for the first year, ninety-five percent for the second year and ninety percent thereafter. *Id.* For those states that implemented the expansion after 2015, it started at ninety percent. *Id.*

66 The law excludes five percent of income from the calculation, so the threshold is effectively 138% of the federal poverty level. *See* ASPE Off. of Health Pol’y, Health Coverage Under the Affordable Care Act: Current Enrollment Trends and State Estimates 2 (Mar. 23, 2023), https://aspe.hhs.gov/sites/default/files/documents/8e81cf90c721dbbf58694c98e85804d3/health-coverage-under-aca.pdf [https://perma.cc/R3SW-FYAT] ("The ACA established a Medicaid eligibility level of 133% of FPL for children, pregnant women, and adults as of January 2014, and included a standard income disregard of five percentage points of the federal poverty level, which effectively raises this limit to 138% FPL Medicaid.”).

67
*See id.* (discussing the expanded eligibility available for adults with incomes below 133% of the FPL).

68 42 U.S.C. § 18051.

69
*Basic Health Program*, Medicaid.gov, https://www.medicaid.gov/basic-health-program/index.html [https://perma.cc/CLB3-BR6B] (last visited Mar. 27, 2021).

70
*History of the Supreme Court Ruling’s Impact on Affordable Care Act Medicaid Expansion Through Federal Fiscal Year 2013*, Kan. Legis. Rsch. Dep’t (Nov. 30, 2023), https://klrd.gov/2023/11/30/supreme-court-rulings-impact-on-affordable-care-act/ [https://perma.cc/2G82-366R].

71 Nat’l Fed’n of Indep. Bus. v. Sebelius, 567 U.S. 519, 584-85 (2012).

72
*Status of State Medicaid Expansion Decisions: Interactive Map*, Kaiser Fam. Found. (Oct. 4, 2023), https://www.kff.org/medicaid/issue-brief/status-of-state-medicaid-expansion-decisions-interactive-map/ [https://perma.cc/23X4-7AK8].

73
*Total Medicaid Enrollment From 1966 to 2022*, Statista, https://www.statista.com/statistics/245347/total-medicaid-enrollment-since-1966/ [https://perma.cc/YAC3-6NUU] (last visited Jan. 23, 2024) (noting that Medicaid covered more than 25 percent of the United States population by 2022); *New Vintage 2021 Population Estimates Available for the Nation, States and Puerto Rico: Estimates Show Slowest Growth on Record for the Nation’s Population*, U.S. Census Bureau, https://www.census.gov/newsroom/press-releases/2021/2021-population-estimates.html [https://perma.cc/W6VG-U3GV] (last visited Jan. 23, 2024) (reporting a total U.S. population of 331,893,745 in 2021).

74 Families First Coronavirus Response Act, Pub. L. 116-127, § 6008, 134 Stat. 178, 208-09 (2020) (temporarily prohibiting states from disenrolling Medicaid beneficiaries during the federally declared COVID-19 Public Health Emergency); *Medicaid Enrollment and Unwinding Tracker*, Kaiser Fam. Found. (Sep. 30, 2025), https://www.kff.org/report-section/medicaid-enrollment-and-unwinding-tracker-overview/#8815e057-6ee9-4945-8ca1-705913d143b8 [https://perma.cc/C7CW-QSJM] (reporting that over twenty-five million Medicaid beneficiaries lost coverage following the end of the COVID-19 PHE and the expiration of the disenrollment freeze).

75
*Total Medicaid Federal and State Expenditures in the United States from 1966 to 2022*, Statista (Dec. 2023), https://www.statista.com/statistics/245350/total-medicaid-federal-and-state-expenditure-in-the-us-since-1966/ [https://perma.cc/4KGB-C3TP]; Cong. Rsch. Serv., R42640, Medicaid Financing & Expenditures 5 fig. 1 (2025).

76
*Annual Expenditures by Insurance Payer*, Medicaid and CHIP Scorecard, https://www.medicaid.gov/state-overviews/scorecard/measure/Annual-Expenditures-by-Insurance-Payer?measure=EX.1&measureView=national&dataView=pointInTime&chart=bar&timePeriods=%5B%222022%22%5D&chartEntities=%5B%22Private%20Health%20Insurance%22,%22Medicare [https://perma.cc/3PCW-QDBJ] (last visited Jan. 23, 2024).

77
*See Health Care Finance Administration*, Fed. Reg., https://www.federalregister.gov/agencies/health-care-finance-administration [https://perma.cc/9PU5-GU6G] (last visited Aug. 19, 2025). The name of the agency was changed from the Health Care Finance Administration in 2001. *Id.*

78 42 U.S.C. § 1315.

79 Elizabeth Hinton, Robin Rudowitz & MaryBeth Musumeci, Kaiser Fam. Found., 3 Key Questions: Section 1115 Medicaid Demonstration Waivers, Issue Brief 3 (2017), https://files.kff.org/attachment/Issue-Brief-3-Key-Questions-Section-1115-Medicaid-Demonstration-Waivers [https://perma.cc/6V2X-2SGT]. In 2005, Congress authorized CMS to go an additional step and offer money to states that implement these arrangements to cover the cost of care outside of institutions. *See* Deficit Reduction Act of 2005, Pub. L. No. 109-171, 120 Stat. 4, 113-20 (2006).

80 Hinton et al., *supra* note 79.

81
*See* Erika Crable et al., *How Do Medicaid Agencies Improve Substance Use Treatment Benefits? Lessons from Three States’ 1115 Waiver Experiences*, 47 J. Health Pol., Pol’y, & L. 497, 497 (2022). In 2015, CMS encouraged states to submit waiver applications to test approaches to expanding treatment. *Id.* The underlying goal was to overcome barriers to care resulting from fragmented care and unstable funding streams. *Id.* In response, states have tested twenty-nine different evidence-based approaches. *See* Erika Crable et al., *Translating Medicaid Policy into Practice: Policy Implementation Strategies from Three US States’ Experiences Enhancing Substance Use Disorder Treatment*, 17 Implementation Sci. 3, 3 (2022). Results have been mixed, but the waiver process enabled policy makers to gain new understanding of what does and does not work. *Id.*

82
*See* 42 U.S.C. § 1396n; Gary Smith et al., U.S. Dep’t of Health & Hum. Servs., Using Medicaid to Support Working Age Adults with Serious Mental Illnesses in the Community: A Handbook 111 (2005), https://aspe.hhs.gov/sites/default/files/migrated_legacy_files//41461/handbook.pdf [https://perma.cc/AL7U-UBPP].

83
*See* Lindsay Shea & Robert I. Field, *Medicaid Coverage for Autistic Individuals: Coverage, Gaps, and Research Needs*, 13 Drexel L. Rev. 961, 985 (2021) (“Such programs will also be important in addressing the needs of their families and caregivers, providers of services for them, and other professionals who assist them, including legal professionals.”).

84
*Id.* at 968.

85
*See generally* State Health Access Data Assistance Ctr., Using 115 Waivers to Fund State Uncompensated Care Pools (2020), https://shadac-pdf-files.s3.us-east-2.amazonaws.com/s3fs-public/publications/Uncompensated%20Care%20Pool_Brief.pdf [https://perma.cc/7TZD-ZSAT](describing the way seven states have used § 1115 waivers to fund uncompensated care at hospitals). Waivers have also been designed to reduce Medicaid coverage, specifically waivers under Section 1115 to impose work requirements on beneficiaries capable of employment. *See* Jane Perkins, *The Administration’s Medicaid Waivers: Exploding in the Guise of Experimenting*, 13 St. Louis U. J. Health L. & Pol’y 53, 59-60 (2019). Only two have gone into effect (in Arkansas and Georgia). Akeiisa Coleman & Sara Federman, *Work Requirements for Medicaid Enrollees*, The Commonwealth Fund (Sep. 30, 2025), https://www.commonwealthfund.org/publications/explainer/2025/sep/work-requirements-medicaid-enrollees [https://perma.cc/JGV5-HNTD]. The Arkansas program resulted in loss of coverage for thousands of beneficiaries who met the waiver’s requirements but failed to comply with reporting requirements. *Id.* (“In Arkansas, more than 18,000 Medicaid enrollees lost coverage in the nine-month period in which the work requirement was in effect. Survey research revealed that the reporting process was a major driver of coverage losses."). *Id.* However, waivers are no longer needed by states to impose work requirements, since they are imposed nationwide by the 2025 budget bill. *See* Elizabeth Hinton, et al., *A Closer Look at the Work Requirement Provisions in the 2025 Federal Budget Reconciliation Law*, Kaiser Fam. Found. (July 30, 2025), https://www.kff.org/medicaid/a-closer-look-at-the-work-requirement-provisions-in-the-2025-federal-budget-reconciliation-law/ [https://perma.cc/HU6X-NEYW].

86
*Program History and Prior Initiatives*, Medicaid.gov, https://www.medicaid.gov/about-us/program-history/index.html [https://perma.cc/VE8W-EUWT] (last visited Oct. 15, 2025). CHIP programs now cover children in families with incomes up to at least 200% of the federal poverty level in all states. *Id.*

87
*CHIP Benefits*, Medicaid.gov, https://www.medicaid.gov/chip/benefits/index.html [https://perma.cc/6N4X-P2HQ] (last visited Mar. 27, 2021).

88
*Program History and Prior Initiatives*, *supra* note 86.

89
*May 2025 Medicaid & CHIP Enrollment Data Highlights*, Medicaid.gov (Apr. 2025), https://www.medicaid.gov/medicaid/program-information/medicaid-and-chip-enrollment-data/report-highlights/index.html [https://perma.cc/Z52Q-F5D7] (reporting Medicaid enrollment at 70,828,934 and CHIP enrollment at 7,266,800 for a total of 78,095,734).

90 Jordan Rau & Emmarie Huetteman, *Urban Hospitals of Last Resort Cling to Life in Time of COVID*, Kaiser Fam. Found.: Health News (Sep. 17, 2020), https://kffhealthnews.org/news/urban-hospitals-of-last-resort-cling-to-life-in-time-of-covid/ [https://perma.cc/39R8-UAVP].

91
*Id.*

92 Dennis P. Andrulis, *The Public Sector in Health Care: Evolution or Dissolution?*, 16 Health Affs. 131, 131 (1997).

93

Dhruv Khullar , Zirui Song & Dave A. Chokshi , *Safety-Net Health Systems at Risk: Who Bears the Burden of Uncompensated Care?*, Health Affs.: Forefront (May 10, 2018), 10.1377/forefront.20180503.138516 (“What happens when a safety-net health system closes? Evidence suggests that the total demand for uncompensated care in a health care market does not change and that there is nearly complete spillover of uncompensated care to remaining hospitals.”).

94 Rau & Huetteman, *supra* note 90.

95 Ian Gavigan & Amy Zanoni, *Hahnemann Shutdown Shows City Hasn’t Learned from Gutting of Philadelphia General Hospital*, Phila. Inquirer (July 18, 2019), https://www.inquirer.com/opinion/commentary/hahnemann-closure-safety-net-public-hospitals-philadelphia-general-hospital-20190718.html [https://perma.cc/GMU9-VJHY].

96 Dave Muoio, *Wellstar’s Atlanta Hospital Closure Has Government Leaders Scrambling to Head Off Care Shortages*, Fierce Healthcare (Sep. 13, 2022, at 12:14 ET), https://www.fiercehealthcare.com/providers/wellstars-atlanta-hospital-closure-has-government-leaders-scrambling-head-care-shortages [https://perma.cc/B7YG-74LY].

97 Dinesh R. Pain, Hengameh Hosseini & Richard S. Brown, *Does Efficiency and Quality of Care Affect Hospital Closures?*, 8 Health Sys. 17, 27 (2019).

98
*Id.*

99
*See id.* at 17; *Fact Sheet: Underpayment by Medicare and Medicaid*, Am. Hosp. Ass’n (Feb. 2022), https://www.aha.org/fact-sheets/2020-01-07-fact-sheet-underpayment-medicare-and-medicaid [https://perma.cc/FFQ7-UN9P] (“Combined underpayments were $100.4 billion in 2020, up from $75.8 billion in 2019. The 2020 underpayment includes a shortfall of $75.6 billion for Medicare and $24.8 billion for Medicaid.”). A study of hospital closures in Pennsylvania between 1999 and 2013 found higher rates of closure among nonprofit than for-profit hospitals, teaching than non-teaching hospitals, and urban than rural hospitals. Pain, *supra* note 97, at 17. The total number of hospitals in the state during this time fell from 170 to 142. *Id.* at 20. The number shrank from 22 to 21 for for-profit and from 148 to 121 for nonprofit hospitals, from 41 to 29 for teaching and from 129 to 113 for non-teaching hospitals, and from 106 to 85 for urban and from 64 to 57 for rural hospitals. *Id.* Of course, many factors were at work other than the level of public support. *See id.* at 17 (discussing the impact of the registered nurse per bed ratio, operating margin, source of revenue, and competition in influencing a hospital’s likelihood to close). Notably, during this time, advances in technology permitted more care to be delivered in outpatient settings and at home, insurance deductibles for hospital care rose, and competition intensified. *Id.* However, the disproportionate impact on hospitals focused on community service and serving more indigent populations suggests that public support played a major role. *Id.*

100 Harold Brubaker, *Philly’s Tough Hospital Market — Not Greed — Did in Hahnemann*, Phila. Inquirer (June 20, 2020), https://www.inquirer.com/business/health/hahnemann-university-hospital-closed-private-equity-freedman-philadelphia-20200620.html [https://perma.cc/7YKF-U39N].

101 Rau & Huetteman, *supra* note 90.

102 Rau & Huetteman, *supra* note 90.

103 U.S. Gov’t Accountability Off., GAO-21-93, Rural Hospital Closures: Affected Residents Had Reduced Access to Health Care Services 1 (2020). Two federal programs supplement Medicaid payments to rural hospitals, the Medicare-dependent Hospital Program and the Low-Volume Hospital Program. *See* Robert King, *Hospitals to Congress: Clock Ticking to Renew Key Rural Hospital Pay Programs*, Fierce Healthcare (Aug. 31, 2022), https://www.fiercehealthcare.com/providers/hospitals-congress-clock-ticking-renew-key-rural-hospital-pay-programs [https://perma.cc/GW54-CHPY].

104 Comm. on the Changing Mkt., Managed Care, and the Future Viability of Safety Net Providers, Inst. of Med., America’s Health Care Safety Net: Intact but Endangered 3 (Marion Ein Lewin & Stuart Altman eds., 2000).

105
*Medicaid Disproportionate Share Hospital (DSH) Payments*, Medicaid.gov, https://www.medicaid.gov/medicaid/financial-management/medicaid-disproportionate-share-hospital-dsh-payments/index.html [https://perma.cc/3ZZ8-HG5M] (last visited Nov. 1, 2022).

106 Cong. Rsch. Serv., R42865, Medicaid Disproportionate Share Hospital Payments 1-2 (2023).

107
*Id.* Base rate payment varies considerably between states. *See* Peter Cunningham et al., Kaiser Fam. Found., Understanding Medicaid Hospital Payments and the Impact of Recent Policy Changes 1-2 (2016).

108 Cong. Rsch. Serv., *supra* note 106, at 2; *see* Omnibus Budget Reconciliation Act of 1981, Pub. L. No. 97-35, 95 Stat. 357, 808-09. DSH payments were implemented in response to a change in Medicaid’s reimbursement rules for hospitals. At the program’s inception in 1965, states were required to pay hospitals the reasonable cost of providing care. *See* Medicare and Medicaid Act of 1965, Pub. L. 89-97, 79 Stat. 286, 354. Most states linked their payment rates to Medicare’s, which were based on the same criterion. *See* Medicaid & CHIP Payment & Access Comm’n, Report to Congress on Medicaid Disproportionate Share Hospital Payments 2 (2016) [hereinafter Report on Disproportionate Share Hospital Payments]. This requirement was removed in 1980, and additional legislation passed the following year permitted states to reimburse hospitals at less than the reasonable cost of care. *See id.* at 3. However, the law directed them to “take into account the situation of hospitals which serve a disproportionate number of low-income patients.” *Id.* In 1987, Congress made these payments mandatory for hospitals that serve the highest share of low-income patients. *See* 42 U.S.C. §§ 1396r-4(b). In 1993, they were set at the difference between the amount of Medicaid reimbursement and the unpaid costs of treating the uninsured. *See id.* at 5.

109 Cunningham et al., *supra* note 107, at 3 (“Nationally, all supplemental Medicaid payments combined amounted to forty-four percent of Medicaid fee-for-service payments to hospitals in 2014 … . Supplemental payments as a proportion of total Medicaid fee-for-service payments to hospitals varies from a low of about 2 percent in North Dakota, South Dakota, and Maine to more than two-thirds in Vermont and Pennsylvania.”).

110
*Disproportionate Share Hospital Payments*, MACPAC (July 26, 2023), https://www.macpac.gov/subtopic/disproportionate-share-hospital-payments/ [https://perma.cc/7S4E-BNU9].

111
*Medicaid Hospital Payment: A Comparison across States and to Medicare*, MACPAC (Apr. 2017), https://www.macpac.gov/wp-content/uploads/2017/04/Medicaid-Hospital-Payment-A-Comparison-across-States-and-to-Medicare.pdf [https://perma.cc/3SNZ-MQXE].

112 Cong. Rsch. Serv., *supra* note 106, at 1.

113 U.S. Gov’t Accountability Off., *supra* note 103.

114
*Id.* Since 1986, safety net hospitals have also received DSH payments through Medicare. *See* Consolidated Omnibus Budget Reconciliation Act (COBRA) of 1985, Pub. L. No. 99-272, § 9105, 100 Stat. 82, 158-60 (1986); 42 C.F.R. § 412.106 (1989). *See also Disproportionate Share Hospital (DSH)*, Ctrs. For Medicare & Medicaid Servs., https://www.cms.gov/medicare/payment/prospective-payment-systems/acute-inpatient-pps/disproportionate-share-hospital-dsh [https://perma.cc/L84A-GY2L] (last visited July 27, 2024).

115 Katherine Neuhausen et al., *Disproportionate Share Hospital Payment Reductions May Threaten Financial Stability of Safety-Net Hospitals*, 33 Health Affs. 988, 989 (2014) (“California’s public safety-net hospitals depend heavily on federal Medicaid Disproportionate Share Hospital (DSH) payments, which are supplemental payments to hospitals that treat large numbers of low-income patients, to offset uncompensated care costs and Medicaid shortfalls.”).

116 Evan S. Cole et al., *Identifying Hospitals That May Be at Most Financial Risk from Medicaid Disproportionate-Share Hospital Payment Cuts*, 33 Health Affs. 2025 (2014). An analysis conducted in 2014 soon after the ACA went into effect found that states that did not expand Medicaid had a larger percentage of hospitals eligible for DHS payments that were in weak financial condition. *Id.*

117 MACPAC, MACStats: Medicaid and CHIP Data Book 9 (2019) (“Disproportionate share hospital (DHS), upper payment limit, and other types of supplemental payments accounted for over half of fee-for-service payments to hospitals in FY2018.”). As an example of the magnitude of federal payments for some states, in 2018, Pennsylvania received $1.5 billion in total Medicaid payments from the federal government, of which $648 million were DSH payments and $396 million were other supplemental payments. *Id.*

118 MACPAC, *supra* note 111, at 1, 8. The report found that Medicaid payments with supplements were higher than Medicare for the conditions studied in twenty-five states and lower in twenty-two, with an average excess over Medicare of six percent. *Id.* at 1. Without supplemental payments, Medicaid rates are often substantially lower than those for Medicare, ranging from an average of seventy percent for heart failure to ninety-two for pulmonary edema. *Id.* at 8.

119 MACPAC, Report to Congress on Medicaid and CHIP 169 (2021).

120 Susan Camilleri, *The ACA Medicaid Expansion, Disproportionate Share Hospitals, and Uncompensated Care*, 53 Health Servs. Rsch. 1562, 1563 (2018).

121
*Id.* at 1563.

122
*Id.* at 1577.

123 David Dranove, Craig Garthwaite & Christopher Ody, *Uncompensated Care Decreased at Hospitals in Medicaid Expansion States but Not at Hospitals in Nonexpansion States*, 35 Health Affs. 1471, 1472 (2016). This study also estimated that uncompensated care costs would have decreased by between 5.7 and 4.0 percent in nonexpansion states had they implemented the expansion. *Id.* A later analysis by the same authors compared hospital financial performance in 2013, just before the expansion, and 2015, a year after it took effect and found a reduction in the overall hospital uncompensated burden from the expansion of between 3.9 and 2.3 percent of operating costs. David Dranove, Craig Garthwaite & Christopher Ody, The Commonwealth Fund, The Impact of the ACA’s Medicaid Expansion on Hospitals’ Uncompensated Care Burden and the Potential Effects of Repeal 1 (2017). Savings across all states were found to total $6.2 billion. *Id.*

124 Dranove et al., *supra* note 123, at 1. Among other research is a study focusing on the years 2016 and 2017 that found a decrease in uncompensated care costs and increase in Medicaid revenue for hospitals in expansion states of all ownership types, although with a larger effect on for-profit than on nonprofit hospitals. *See* Fredric Blavin & Christal Ramos, *Medicaid Expansion: Effects on Hospital Finances and Implications for Hospitals Facing COVID-19 Challenges*, 40 Health Affs. 82, 87 (2021).

125 Richard C. Lindrooth et al., *Understanding the Relationship Between Medicaid Expansions and Hospital Closures*, 37 Health Affs. 111, 115-16 (2018).

126 Am. Hosp. Ass’n, Rural Hospital Closures Threaten Access: Solutions to Preserve Care in Local Communities 7 (2022).

127
*Id.* at 5.

128 U.S. Gov’t Accountability Off., GAO-21-341SP, Medicaid in Times of Crisis 1 (2021) (“When pandemics, economic recessions, natural disasters, and other crises occur, states and the federal government initiate efforts to mitigate damage and protect vulnerable groups. Some of these efforts have included using Medicaid to ensure eligible individuals continue to have access to essential health services.”). Among other research findings, a 2018 study found that Medicaid was associated with increases in coverage, service use, and quality of care. *See* Olena Mazurenko et al., *The Effects of Medicaid Expansion under the ACA: A Systematic Review*, 37 Health Affs. 944, 944 (2018). A 2017 study found that it was associated with improved quality of care at federally funded health centers. *See* Megan B. Cole et al., *At Federally Funded Health Centers, Medicaid Expansion was Associated with Improved Quality of Care*, 36 Health Affs. 40, 47 (2017). A 2020 study found that Medicaid was associated with improved health among the near-elderly. *See* Melissa McInerney et al., *ACA Medicaid Expansion Associated with Increased Medicaid Participation and Improved Health Among Near-Elderly: Evidence from the Health and Retirement Study*, 57 J. Health Care Org., Provision, & Fin. 1, 2-3 (2020). And several studies have found that it improved the financial health of hospitals in states that accepted it. *See* Blavin & Ramos, *supra* note 124 (finding early positive financial effects of the expansion on hospital finances that continued in 2016 and 2017 due to decreased uncompensated care and increased Medicaid revenue that produced increased financial margins).

129
*Skilled Nursing Facility Care*, Medicare.gov, https://www.medicare.gov/coverage/skilled-nursing-facility-care [https://perma.cc/W9F7-2L57] (last visited Sep. 1, 2025). Medicare is also an important source of funding for nursing home care, but its coverage is limited to one-hundred days in a skilled nursing facility immediately following an acute care hospital stay as opposed to extended stays for those who need ongoing assistance for an indefinite period of time. *Id.* Medicaid coverage for institutional care applies to several different kinds of facilities, including skilled nursing, rehabilitation, less intensive long-term care, and intermediate care for patients with intellectual disability. *Nursing Facilities*, Medicaid.gov, https://www.medicaid.gov/medicaid/long-term-services-supports/institutional-long-term-care/nursing-facilities [https://perma.cc/U9T5-Z88W] (last visited Oct. 17, 2025).

130
*Medicaid’s Role in Nursing Home Care*, Kaiser Fam. Found. (June 20, 2017), https://www.kff.org/infographic/medicaids-role-in-nursing-home-care/ [https://perma.cc/VS55-RQC7].

131
*Id.* The total Medicaid budget in 2020 was $597.6 billion. *Long Term Services & Supports*, Medicaid.gov, https://www.medicaid.gov/medicaid/long-term-services-supports/index.html [https://perma.cc/TC5W-DP2Y] (last visited Jan. 29, 2024).

132
*Medicaid’s Role in Nursing Home Care*, *supra* note 130. Private long-term care insurance is available to help cover the cost, but only 7.5 million Americans have purchased such coverage. *7.5 Million Americans Have Long-Term Care Insurance Protection*, Am. Ass’n for Long-Term Care Ins. (Jan. 14, 2020), https://www.aaltci.org/news/long-term-care-insurance-news/7-5-million-americans-have-long-term-care-insurance-protection [https://perma.cc/W8JS-D63A].

133 Henry Brodaty & Marika Donkin, *Family Caregivers of People with Dementia*, 11 Dialogues in Clinical Neuroscience 217, 222 (2009).

134
*Id.* at 217.

135
*Id.* at 219-20.

136 Lynn Feinberg et al., *Valuing the Invaluable: 2011 Update: The Growing Contributions and Costs of Family Caregiving*, AARP Pub. Pol’y Inst. 1 (2011), https://www.beliveaulaw.net/wp-content/uploads/2011/08/AARPs-Valuing-the-Invaluable-2011-Update-The-Growing-Contributions-and-Costs-of-Family-Caregiving.pdf [https://perma.cc/KFN2-QL5G].

137 Daniel W. L. Lai, *Effect of Financial Costs on Caregiving Burden of Family Caregivers of Older Adults*, Sage Open, Oct.–Dec. 2012, at 1.

138
*Sharing Caregiving Responsibilities*, Nat’l Inst. on Aging (Oct. 12, 2023), https://www.nia.nih.gov/health/caregiving/sharing-caregiving-responsibilities [https://perma.cc/RD87-PTRM] (“About one in four caregivers care for their children as well as an aging parent or partner. These caregivers are referred to as the ‘sandwich generation.’ Sandwich generation caregivers may face additional emotional and financial challenges in caring for both children and parents.”).

139
*See generally* Milton Friedman with the assistance of Rose D. Friedman, Capitalism and Freedom (40th anniversary ed. 2002) (presenting an argument in favor of *laissez faire* economics).

140
*See generally* Adam Smith, An Inquiry into the Nature and Causes of the Wealth of Nations 49 (Sálvio Soares ed., MetaLibri Digital Library 2007) (1776) (ebook) (arguing that exchanging the different productions of different sorts of labor for one another are adjusted “not by any accurate measure, but by the haggling and bargaining of the market, according to that sort of rough equity which, though not exact, is sufficient for carrying on the business of common life.”).

141 Friedman, *supra* note 139, at 191 (“I am distressed by the sight of poverty; I am benefited by its alleviation; but I am benefited equally whether I or someone else pays for its alleviation.”). Friedman discusses poverty relief as a nonexcludable good that is suboptimally supplied by private individuals in the free market, but a good that nonetheless benefits both the poor and nonpoor. *Id.*

142 Sherman Folland et al., The Economics of Health and Healthcare 266-67, 392 (Pearson/Prentice Hall 7th ed. 2013). *See also* Francis M. Bator, *The Anatomy of Market Failure*, 72 Q.J. Econ. 351, 351 (1958) (defining “market failure” as the failure of price-market institutions to sustain “desirable activities” or stop “undesirable activities”).

143
*Public Goods*, Federal Reserve Education https://www.federalreserveeducation.org/teaching-resources/economics/role-of-government/public-goods [https://perma.cc/9A5F-T2ES] (last visited Aug. 26, 2025).

144 Folland et al., *supra* note 142, at 283.

145 Nigar Hashimzade et al., *Public Good*, *in* A Dictionary of Economics (5th ed., 2017). The concept of public goods was first described by economist Paul Samuelson in 1954. *See* Paul Samuelson, *The Pure Theory of Public Expenditure*, 36 Rev. Econ. Stat. 387, 387 (1954).

146
*See* Folland et al. *supra* note 142. Another example is light from a lighthouse, which was essential for maritime navigation before GPS technology was developed. It is nonexclusive in that the lighthouse operator cannot prevent nonpaying ships from using it, and it is nonrival in that guidance by one ship does not dimmish the supply of light for others. *See* Theresa Levitt, *When Lighthouses Became Public Goods: The Role of Technological Change*, 61 Tech. & Culture 144, 161 (2020).

147
*See generally* Robert Baldwin et al., Understanding Regulation: Theory, Strategy, and Practice. Oxford University Press 15-25 (2d ed. 2013) (discussing market influences and the likelihood of public goods to fail due to overconsumption).

148 W. Ver Eecke. *Public Goods: An Ideal Concept*, 28 J. Socio-Econ. 139, 140 (1999) ("The concept of public goods has validity because it points to an opportunity for gain if collective action can be taken.”).

149
*See* Leonard Champney, *Public Goods and Policy Types*, 48 Public Admin. Rev. 988, 989-90 (1988) (describing types of government policies that can be used to facilitate the production of public goods).

150
*Id.* at 989.

151
*See id.* at 990 (stating “government routinely regulates incorporated economic institutions. For example, the state attempts to produce the public good of environmental quality by compelling corporations to cease discharge of pollutants into the air and water.”).

152
*Id.* (“Distributive policy directly or indirectly offers rewards to corporations for activities that lead to the production of public goods or assumes the costs of these activities. Weapons procurement provides one example, as does subsidization of corporate activities deemed appropriate to the production of a clean environment.”).

153
*See id.* at 991 (describing “policies designed to *produce* public goods directly”).

154
*Id.*

155 Emma Harden-Wolfson & Jose Antonio Quinteiro, *Public Goods, Common Goods and Global Common Goods: A Brief Explanation*, UNESCO (Dec. 6, 2024), https://www.iesalc.unesco.org/en/2022/04/10/public-goods-common-goods-and-global-common-goods-a-brief-explanation/ [https://perma.cc/DF48-FVBX].

156
*Id.*

157
*See id.*

158
*Universal Health Insurance Is a Common Good*, Economist (Oct. 8, 2009), https://www.economist.com/democracy-in-america/2009/10/08/universal-health-insurance-is-a-common-good [https://perma.cc/39EF-CYL3]. While systems of universal health care exist in most developed countries, the United States does not have one. *See* Bruce Vladeck, *Universal Health Insurance in the United States: Reflections on the Past, the Present, and the Future*, 93 Am. J. Pub. Health 16, 16 (2003).

159 William A. McEachern, Economics: A Contemporary Introduction 341 (2003) (defining “quasi-public goods” as “[a] good that is nonrival but exclusive is called a quasi-public good”).

160
*Id.* A similar concept is that of “club goods," which are produced by voluntary associations of individuals for their mutual benefit. Bruce L. Benson, *Are Roads Public Goods, Private Goods, Club Goods, or Common Pools*?, *in* Explorations in Public Sector Economics 174 (Joshua Hall ed., 2017). For example, members of a professional may form a professional association to monitor the quality of care rendered by its members. *See id.* “[A] club good differs from a private good in that it ‘belongs’ to and is used by a limited voluntary association of decision-makers who face collective decision-making costs.” *Id.*

161
*Id.* at 171, 207.

162 Thalia Gonzalez & Giovanni Saarman, *Regulating Pollutants, Negative Externalities, and Good Neighbor Agreements: Who Bears the Burden of Protecting Communities?*, 41 Ecology L. Q. 37, 39 (2014) (“This structure allows market mechanisms to impose the burden on communities to mitigate the negative externalities of pollution by engaging in environmental policing.”).

163
*Id.*

164
*See* Emma Hutchinson, Principles of Microeconomics, 289-301 (2017). “Because externalities that occur in market transactions affect other parties beyond those involved, they are sometimes called spillovers.” *Id.* at 293.

165
*See id.*

166
*Spillover Effect*, Corp. Fin. Inst., https://corporatefinanceinstitute.com/resources/economics/spillover-effect/ [https://perma.cc/79UA-JQ8P] (last visited Jan. 23, 2024).

167 Gonzalez & Saarman, *supra* note 162, at 39.

168 Timothy Besley & Stephen Coate, *Public Provision of Private Goods and The Redistribution of Income*, 81 Am. Econ. Rev. 979, 979 (1991) (“Most governments devote considerable resources to the provision of private goods such as health, housing, and education.”).

169
*See* Mark A. Hall & Judith Garber, *Exclusion of Elective Care from Hospital Financial Assistance Policies — Arresting a Troubling Development*, 393 New Eng. J. Med. 107, 108-09 (2025). As a general rule, hospitals can prospectively deny access to beds for elective care for patients who cannot demonstrate the ability to pay, generally by providing evidence of insurance. *Id.*

170
*See* Gaby Galvin, *For-Profit Insurers Poised to Benefit if Democrats Succeed at Plugging Medicaid Coverage Gap*, Morning Consult (July 21, 2021), https://morningconsult.com/2021/07/21/medicaid-expansion-for-profit-insurers-business/ [https://perma.cc/DWS7-V3T7]. Industries other than hospitals also benefit from Medicaid. Among the most significant is the insurance industry. Forty-one states rely on private insurance companies to administer Medicaid benefits through managed care to all least some of its beneficiaries, in many of those states to most. *Medicaid Managed Care 101*, Nat’l Conf. of State Legislatures (Sep. 21, 2023), https://www.ncsl.org/health/medicaid-managed-care-101 [https://perma.cc/4MNM-KAP7] (map shows percentage of Medicaid enrollees covered by managed care in each states). Some that serve this market rely on Medicaid for most of their business. For example, Molina derives 89.3 percent of its enrollment from Medicaid and Centene derives 53.3 percent. *Id.* As Medicaid enrollment grows, so do the fortunes of these companies. When the COVID pandemic drove more people to Medicaid for health care coverage, Molina saw a 21.8 percent jump in enrollment and Centene a 56.7 percent jump. *Id.* Other companies seeing increases in enrollment for their Medicaid line of business included Aetna at 39.2 percent, Anthem at 21.8 percent, and UnitedHealth at 12.2 percent. *Id.*

171
*See* Saroush Saghafian et al., *Towards a More Efficient Healthcare System: Opportunities and Challenges Caused by Hospital Closures Amid the COVID-19 Pandemic.* 25 Health Care Mgmt. Sci. 187, 187 (2022).

172
*See* Abigail Silva, *Where You Live Matters to Your Health*, 8 AMA J. Ethics 766, 766-67 (2006).

173 Reed Abelson, *Covid Overload: U.S. Hospitals Are Running Out of Beds for Patients*, N.Y. Times (Nov. 27, 2020), https://www.nytimes.com/2020/11/27/health/covid-hospitals-overload.html [https://perma.cc/HZM3-LWZ7].

174
*See* Sue Anne Bell et al., *All-Cause Hospital Admissions Among Other Adults After a Natural Disaster*, 71 Annals Emergency Med. 746, 753 (2018) (finding that all-cause hospital admissions increased in the thirty days following a major hurricane and concluding “[t]he results of this study point to the need for building community and health care system resilience to account for health care needs of older adults after a disaster”).

175 Pain et al., *supra* note 97.

176 Renee Y Hsia, Arthur L. Kellermann & Yu-Chu Shen, *Why Are Many Emergency Departments in the United States Closing?*, Rand Health (Sep. 9, 2011), https://www.rand.org/pubs/research_briefs/RB9607.html [https://perma.cc/RT9N-DX4Z] [hereinafter *Why Are Many Emergency Departments in the United States Closing?*].

177 42 U.S.C § 1395dd (2024).

178 Joseph Zibulewsky, *The Emergency Medical Treatment and Active Labor Act (EMTALA): What It Is and What It Means for Physicians*, 14 Baylor Univ. Med. Ctr. Proc. 339, 340-43 (2017).

179 EMTALA also does not establish a standard for quality of care. *See* Baber v. Hosp. Corp. of Am., 977 F.2d 872, 878 (4th Cir. 1992). Courts have denied recovery for substandard care, if there was no discrimination between paying and nonpaying patients. *Id.*

180 Although EMTALA requires that emergency care be provided to all who present themselves, emergency departments can become overcrowded. *See* Chung-Yao Kao, Jhen-Ci Yang & Hin-Hao Lin, *The Impact of Ambulance and Patient Diversion on Crowdedness of Multiple Emergency Departments in a Region.* PLOS-One 10, at 1 (2015) (“Ambulance diversion (AD) may relieve ED overcrowding; however, diverting patients from an overcrowded ED will load neighboring EDs with more patients and may result in regional overcrowding.”). *See* Marina Sartini et al., Overcrowding in Emergency Department: Causes, Consequences, and Solutions A Narrative Review, 10 Healthcare 1625 (2022), 10.3390/healthcare10091625 PMC949866636141237. In this situation, emergency care has the qualities of a rival good, even if government action has made it is nonexclusive.

181
*See generally* Baber, 977 F.2d at 878-79 (discussing requirements for hospitals under EMTALA). The law has important limitations. Hospitals need only treat patients within the scope of their capabilities and can transfer patients to other facilities if additional resources are needed. 42 U.S.C § 1395dd(b)(1). A patient can be discharged without additional care if the hospital determines after an assessment that he or she does not have an emergency condition or has had one and been stabilized. *See* Baber, 977 F.2d at 884-85.

182 Ryan M. McKenna et al., *Examining EMTALA in the Era of the Patient Protection and Affordable Care Act*, 5 AIMS Pub. Health 366, 368 (2018) (stating patients receiving care under EMTALA “may still be billed after receiving care. This could result in bad debt for both the consumer (i.e. bankruptcy) and the provider (i.e., uncompensated care)”).

183
*Id.*

184
*See Medicaid for the Treatment of an Emergency Medical Condition Fact Sheet*, N.Y. State Dep’t of Health. (Mar. 2021), https://www.health.ny.gov/health_care/medicaid/emergency_medical_condition_faq.htm [https://perma.cc/6X3D-7E9P].

185 Zibulewsky, *supra* note 178, at 346.

186 Renee Y. Hsia et al., *Factors Associated with Closures of Emergency Departments in the United States*, 305 [J]AMA. Ass’n 1978, 1978 (2011) [hereinafter *Factors Associated with Closures of Emergency Departments in the United States*].

187
*Id.* at 1980.

188
*Id.* at 1984.

189
*See* Sarah I. Kamel et al., *Impact of the Closure of a Large, Urban Safety-Net Hospital on a Neighboring Academic Center: A Philadelphia Case Study*, 17 Health Servs. Rsch. & Pol’y 1123, 1123 (2020), 10.1016/j.jacr.2020.04.001 32389550. When Hahnemann Hospital closed, the closest emergency department was operated by Thomas Jefferson University Hospital, located about a mile away. *See id.* In the two months after Hahnemann closed, Jefferson experienced an increase in the number of emergency department patients of more than twenty percent and an overall increase in patient volume of twenty-three percent. *Id.* at 1124. As the number of patients at Jefferson’s emergency department increased, the median time from patient registration to evaluation grew by eighty-two percent as compared to the months before the closure. *Id.*

190 Jesse M. Pines et al., *The Association Between Emergency Department Crowding and Adverse Cardiovascular Outcomes in Patients with Chest Pain*, 16 Acad. Emerg. Med. 617, 623 (2009) (“At our institution, there was an association between higher rates of adverse cardiovascular outcomes and some measures of higher ED crowding.”).

191
*See* Thomas C. Buchmueller et al., *How Far to the Hospital? The Effect of Hospital Closures on Access to Care*, 25 J. Health Econ. 740, 740 (2006).

192
*See* David C. Lee et al., *The Impact of Hospital Closures and Hospital and Population Characteristics on Increasing Emergency Department Volume: A Geographic Analysis*, 18 Population Health Mgmt. 459, 461 (2015), 10.1089/pop.2014.0123 PMC531943125658768. A study of hospitals with emergency departments in New York State between 2004 and 2010 found that those most likely to close were in counties with the highest levels of uninsurance. *Id.*

193

Cheryl Heiks & Nicole Sabine , *Long Term Care and Skilled Nursing Facilities*, 8 Del. J. Pub. Health 144, 144-45 (2022), 10.32481/djph.2022.12.032 PMC989402936751604 .

194
*See What is Nursing Home Level of Care & Its Importance to Medicaid Eligibility*, Am. Council on Aging (June 2, 2023), https://www.medicaidplanningassistance.org/nursing-home-level-of-care/ [https://perma.cc/9FA7-DYUK].

195
*Id.*

196
*See* Lauren Harris-Kojetin et al., Nat’l Ctr. for Health Stat., U.S. Dep’t Of Health & Hum. Servs., No. 43, Long-Term Care Providers and Services Users in the United States, 2015-2016 3 (2019), https://www.cdc.gov/nchs/data/series/sr_03/sr03_43-508.pdf [https://perma.cc/6DNP-T7EX].

197
*Id.* at 1.

198
*Id.*

199
*Id.*

200
*See* Manuel Eskildsen & Thomas Price, *Nursing Home Care in the USA*, 9 Geriatrics & Gerontology Int’l 1, 5 (2009).

201
*Id.*

202 Ctrs. for Medicare & Medicaid Servs., CMS Product No. 10153, Medicare Coverage of Skilled Nursing Facility Care (2019), https://www.medicare.gov/Pubs/pdf/10153-Medicare-Skilled-Nursing-Facility-Care.pdf [https://perma.cc/SVH2-RDUV].

203
*Medicaid Coverage of Nursing Home Care: When, Where and How Much They Pay*, Am. Council on Aging (Nov. 22, 2024), https://www.medicaidplanningassistance.org/medicaid-and-nursing-homes/ [https://perma.cc/DV7D-NE86].

204 Pryia Chidabaram & Alice Burns, *How Many People Use Medicaid Long-Term Services and Supports and How Much Does Medicaid Spend on Those People?*, fig. 1, Kaiser Fam. Found. (Aug. 14, 2023), https://www.kff.org/medicaid/issue-brief/how-many-people-use-medicaid-long-term-services-and-supports-and-how-much-does-medicaid-spend-on-those-people/ [https://perma.cc/FP4D-8NXK].

205
*See Medicaid’s Role in Nursing Home Care*, Kaiser Fam. Found. (June 20, 2017), https://www.kff.org/infographic/medicaids-role-in-nursing-home-care/ [https://perma.cc/2JNW-B33D].

206
*Id.*

207
*Id.*

208
*See id.* However, to qualify for Medicaid, beneficiaries must have incomes that fall below the thresholds specified in each state and have limited financial assets. *Medicaid Eligibility: 2025 Income, Asset & Care Requirements for Nursing Homes & Long-Term Care*, Am. Council on Aging (Jan. 20, 2025), https://www.medicaidplanningassistance.org/medicaid-eligibility/ [https://perma.cc/D667-U3Y8].

209
*See How Many Skilled Nursing Facilities Are in the U.S.?*, Definitive Healthcare (Aug. 1, 2025), https://www.definitivehc.com/resources/healthcare-insights/skilled-nursing-facilities-us#:~:text=How%20many%20beds%20does%20the,average%20of%20around%20116%20beds [https://perma.cc/U8YY-53FG] (reporting that the average number of beds in skilled nursing facilities in the United States in 2025 was 115); *What Is The Average Number of Beds in a U.S. Hospital?*, Definitive Healthcare, (Jan. 15, 2025), https://www.definitivehc.com/resources/healthcare-insights/us-hospitals-average-beds [https://perma.cc/9S32-BW79] (reporting that the average number of beds in short-term acute care hospitals was 187).

210 David R. Zimmerman, *Improving Nursing Home Quality of Care Through Outcomes Data: The MDS Quality Indicators*, 18 Int’l J. Geriatric Psychiatry 250, 250 (2003).

211
*See* Mei-Lan Chen, *The Growing Costs and Burden of Family Caregiving of Older Adults: A Review of Paid Sick Leave and Family Leave Policies*, 56 Gerontologist 391, 391-92 (2014).

212
*See* Deborah Majerovitz, *Predictors of Burden and Depression Among Nursing Home Family Caregivers*, 11 Aging & Mental Health 323, 326-27 (2006).

213
*See* Stuart M. Butler, *Achieving an Equitable National Health System for America*, Brookings (Dec. 9, 2020), https://www.brookings.edu/articles/achieving-an-equitable-national-health-system-for-america/ [https://perma.cc/LCX9-J9MQ].

214 Folland et al., *supra* note 142, at 383-84.

215
*See* Genevieve P. Kanter, Andrea G. Segal & Peter W. Groeneveld, *Income Disparities in Access to Critical Care Services*, 39 Health Affs. 1362, 1362 (2020) (stating that forty-nine percent of the lowest-income communities had no ICU beds in their communities, whereas only 3 percent of the highest-income communities had no ICU beds. Income disparities in the availability of community ICU beds were more acute in rural areas than in urban areas.).

216 Paul Starr, The Social Transformation of American Medicine 373 (1982) (discussing the impact of social programs that aimed to reduce the exclusion from medical care of the poor).

217
*Id.*

218
*Id.*

219
*Id.*

220
*Id.*

221
*Id.*

222
*Id.*

223
*See id.*

224
*See* Sarah Miller & Laura R. Wherry, *Health and Access to Care During the First 2 Years of the ACA Medicaid Expansions*, 376 New Eng. J. Med. 947, 947 (2017) (discussing relative rates of uninsurance and Medicaid participation in states that underwent Medicaid expansion versus those that did not).

225
*Id.*

226 Visla Curto & Monica Bhole, *Impacts of Early ACA Medicaid Expansions on Physician Participation*, 57 Health Serv. Rsch. 881, 881 (2021).

227
*See* Sarah Miller et al., *Medicaid and Mortality: New Evidence from Linked Survey and Administrative Data*, 136 Q. J. Econ. 1783, 1823-24 (2021) (expanding the survey’s estimated change in mortality resulting from ACA Medicaid expansion).

228
*See* Latoya Hill et al., *Health Coverage by Race and Ethnicity, 2010 - 2023*, Kaiser Fam. Found. (Feb. 13, 2025), https://www.kff.org/racial-equity-and-health-policy/health-coverage-by-race-and-ethnicity/ [https://perma.cc/S99J-FSAW] (reporting lower rates of private insurance for Black Americans).

229
*See id.*

230
*Id.* at fig. 2.

231
*Id.*

232
*See* Elizabeth Hinton & Amaya Diana, *Medicaid Authorities and Options to Address Social Determinants of Health*, Kaiser Fam. Found. (Jan. 29, 2024), https://www.kff.org/medicaid/medicaid-authorities-and-options-to-address-social-determinants-of-health-sdoh/ [https://perma.cc/LNQ3-V25M] (explaining that states can use waivers to “to add certain non-clinical services to the Medicaid benefit package including case management, housing supports, employment supports, and peer support services”).

233
*How States Use Federal Medicaid Authorities to Finance Housing-Related Services*, Nat’l Acad. for State Health Pol’y (Mar. 8, 2021), https://nashp.org/how-states-use-federal-medicaid-authorities-to-finance-housing-related-services/ [https://perma.cc/H6PR-KBRV] (discussing the use of 1115 waivers to address housing needs and noting “[t]rends across housing-related 1115 waivers show that states target different groups, but primarily focus on individuals with high emergency department use, SUDs, and serious mental illness (SMI)”).

234
*See* Aksash Pillai et al., *Medicaid Efforts to Address Racial Health Disparities*, Kaiser Fam. Found. (July 1, 2024), https://www.kff.org/medicaid/medicaid-efforts-to-address-racial-health-disparities/ [https://perma.cc/93NY-KRK5] ("States may also use 1115 waivers to expand Medicaid eligibility, add or enhance benefits, or make delivery system changes to address disparities and advance equity.”).

235
*Historic Health Equity Waiver’s First Year*, Mass. Health & Hosp. Ass’n. (Jan. 2, 2024) https://www.mhalink.org/mondayreport/historic-health-equity-waivers-first-year/ [https://perma.cc/4E2Y-VPJG] (discussing Massachusetts’s use of the Section 1115 waiver to increase funding to collect data regarding health disparities and the expansion of this waiver).

236
*Vermont Global Commitment to Health Section 1115 Demonstration Extension* Approval, CMS (June 28, 2022), https://www.cms.gov/newsroom/press-releases/vermont-global-commitment-health-section-1115-demonstration-extension-approval [https://perma.cc/2AK4-SJQP] (discussing the goals and methods of Vermont’s managed care-like use of Section 1115 waivers, including advanced data collection).

237 Sara Rosenbaum, *A Program for All Seasons*, 13 St. Louis U. J. Health L. & Pol’y 5, 15 (2019).

238 Pillai et al., *supra* note 234 (“Most states that contract with managed care plans report leveraging MCO contracts to promote at least one strategy to address social determinants of health in FY 2023, which may help mitigate racial health disparities.”); *see also* Kevin H. Nguyen et al., *Racial and Ethnic Disparities in Patient Experience of Care Among Nonelderly Medicaid Managed Care Enrollees*, 41 Health Affs. 256, 256 (2022) (discussing existing disparities in treatment among managed care organizations and the availability of data to mitigate inequities).

239 Managed Care State Quality Strategy, 42 C.F.R. § 438.340 (2024).

240 Pillai et al., *supra* note 234.

241 Pillai et al., *supra* note 234.

242 Jack Hoadley, Karina Wagnerman, Joan Alker & Mark Holmes, Medicaid in Small Towns and Rural America: A Lifeline for Children, Families, and Communities 3 (2017), https://ccf.georgetown.edu/wp-content/uploads/2017/06/Rural-health-final.pdf [https://perma.cc/59C8-F9V7] (discussing the higher rate of Medicaid enrollment in rural versus urban areas).

243
*Id.*

244
*Id.* at 5.

245

Chiquita Brooks-LaSure & Daniel Tsai ., A Strategic Vision for Medicaid and The Children’s Health Insurance Program (CHIP), Health Affs.: Forefront (Nov. 16, 2021), 10.1377/forefront.20211115.537685 .

246
*See* Naomi Zewde et al., *Improving Estimates of Medicaid’s Effect on Poverty: Measures and Counterfactuals*, 56 Health Servs. Rsch. 1190, 1190 (2021).

247
*Id.*

248
*Id.*

249 George M. Holmes et al., *The Effect of Rural Hospital Closures on Community Economic Health*, 41 Health Servs. Rsch. 467, 467 (2006).

250
*Id.* at 478, tbl. 3.

251
*Id.* The authors of this study suggested that rural hospitals faced particular financial pressure from Medicare’s switch to a prospective payment system of hospital reimbursement in the 1980s, noting the link between government reimbursement and hospital survival. *Id.*

252

Hannah Shadowen et al., *Virginia Medicaid Expansion: New Members Report Reduced Financial Concern During the COVID-19 Pandemic*, 41 Health Affs. 1078, 1078 (2022), 10.1377/hlthaff.2021.01910 35858118.

253
*Id.* at 1078 (“Medicaid is a critical antipoverty program.).

254 Samantha Artiga & Elizabeth Hinton, *Beyond Health Care: The Role of Social Determinants in Promoting Health and Health Equity*, Kaiser Fam. Found. (May 10, 2018), https://www.kff.org/racial-equity-and-health-policy/issue-brief/beyond-health-care-the-role-of-social-determinants-in-promoting-health-and-health-equity/ [https://perma.cc/6X5V-89JN].

255
*See* U.S. Dep’t of Health & Human Servs., Off. of Disease Prevention & Health Promotion, *Poverty*, Healthy People 2030, https://health.gov/healthypeople/priority-areas/social-determinants-health/literature-summaries/poverty [https://perma.cc/AN4Y-7286] (last visited Sep. 14, 2024) (listing several community benefits from reducing poverty).

256
*See* U.S. Dep’t of Health & Human Servs., *supra* note 255 (discussing the impact of childhood poverty on education, nutritional deficits, developmental delays, toxic stress, and chronic illness).

257
*See* Benoît de Courson & Daniel Nettle, *Why do Inequality and Deprivation Produce High Crime and Low Trust?* 11 Nature Sci. Reps. 1937, 1937 (2021) (discussing the relation of crime to the distribution of economic resources).

258 Madalyn Atkins, *How the Business Community Can Help Alleviate Poverty*, ADEC ESG (Apr. 4, 2017), https://www.adecesg.com/resources/blog/how-the-business-community-can-help-alleviate-poverty/#:~:text=The%20effects%20of%20poverty%20in%20our%20communities,makes%20it%20harder%20for%20businesses%20to%20thrive [https://perma.cc/FKC8-9R95] ("The effects of poverty in our communities aren’t theoretical. They show up in reduced productivity, workforce churn, fragile supply chains, and markets that never quite reach their potential. In other words, poverty makes it harder for businesses to thrive.”).

259
*Medicaid Managed Care Spending in 2019*, Healthcare Mgmt Ass’n (May 15, 2020), https://www.healthmanagement.com/blog/medicaid-managed-care-spending-in-2019/ [https://perma.cc/QQ88-3VBC]/

260
*Id.*

261 MACPAC, MACStats: Medicaid and CHIP Data Book 9 (2022), https://www.macpac.gov/wp-content/uploads/2022/12/MACSTATS_Dec2022_WEB-508.pdf [https://perma.cc/D32K-Y4MB].

262
*See* Darcey J. McMaughan, Oluyomi Oloruntoba & Matthew L. Smith, *Socioeconomic Status and Access to Healthcare: Interrelated Drivers for Healthy Aging*, 8 Frontiers Pub. Health, no. 231, June 2020, at 1, 5 (showing that substantial evidence exists to support the strong interplay between SES, health care access, and healthy aging).

263
*See* Ronald Loeppke et al., *Health and Productivity as a Business Strategy: A Multiemployer Study*, 51 J. Occupational & Env’t Med. 411, 411 (2009) (stating that the health-related productivity costs of illness have been found to be larger than direct medical and pharmacy costs).

264
*See* Ron Z. Goetzel et al., *Health, Absence, Disability, and Presenteeism Cost Estimates of Certain Physical and Mental Health Conditions Affecting US Employers*, 46 J. Occupational & Env’t Med. 398, 398 (2004) (discussing the costs of absenteeism due to certain chronic conditions).

265
*Id.* (noting that chronic conditions that are especially likely to reduce worker productivity include depression, anxiety, obesity, arthritis, and back pain).

266
*See* Rebecca J. Mitchell & Paul Bates, *Measuring Health-Related Productivity Loss*, 14 Population Health Mgmt. 93, 97 (2011) (expanding upon estimations of productivity differences between employees with or without health conditions).

267 Christina Robinson & Nicole M. Coomer, *Children’s Public Health Insurance and Maternal Absenteeism*, 39 J. Econ. Insight, no. 2, 2013, at 1.

268 An illustration of the importance of healthy workforces to employers is the prevalence of workplace wellness programs. Four out of five large employers have implemented them. *See* Gary Claxton et al., Kaiser Fam. Found., Employer Health Benefits 2018 Annual Survey 188 (2018), http://files.kff.org/attachment/Report-Employer-Health-Benefits-Annual-Survey-2018 [https://perma.cc/7SYC-Z4HM]. The primary goal is to reduce the incidence of chronic conditions that can adversely affect productivity and to help workers who have them manage their conditions more effectively. Jean Marie Abraham, *Employer Wellness Programs — A Work in Progress*, 321 [J]AMA 1462, 1462 (2019).

269
*See* Hugh Waters & Marlon Graf, Milken Inst., The Cost of Chronic Disease in the U.S. Executive Summary 5 (2018), https://milkeninstitute.org/sites/default/files/reports-pdf/Chronic-Disease-Executive-Summary-r2.pdf [https://perma.cc/JY22-22TB] (discussing the costs, both direct and indirect, of chronic diseases in the United States).

270
*2021 Health Benefits Survey*, Kaiser Fam. Found. (Nov. 10, 2021), https://www.kff.org/report-section/ehbs-2021-section-2-health-benefits-offer-rates/ [https://perma.cc/U4CE-2FCE] (“Firms not offering health benefits continue to cite cost as the most important reason they do not do so.”).

271 Jennifer Tolbert et al., *Understanding the Intersection of Medicaid and Work: An Update*, Kaiser Fam. Found. (May 30, 2025), https://www.kff.org/report-section/work-among-medicaid-adults-implications-of-economic-downturn-and-work-requirements-issue-brief/ [https://perma.cc/32UU-AM9F] (Stating that in 2023, forty-four percent of Medicaid recipients were working full-time and twenty percent were working part-time (figure 1) and that of these, “nearly five in ten (forty-six percent) Medicaid workers were employed in firms with fewer than fifty employees, which are not subject to ACA penalties for not offering affordable health coverage”).

272
*Fast Facts on U.S. Hospitals, 2025*, Am. Hosp. Ass’n, https://www.aha.org/statistics/fast-facts-us-hospitals [https://perma.cc/545T-PRTZ] (last visited Oct. 6, 2025).

273
*Id.*

274 Matt McGough et al., *How Much is Health Spending Expected to Grow?*, Peterson-KFF Health Sys. Tracker (Aug. 4, 2025), https://www.healthsystemtracker.org/chart-collection/how-much-is-health-spending-expected-to-grow/#Total%20health%20spending,%20by%20service%20type,%202018-2023;%20projected%202024-2033 [https://perma.cc/5M33-AA8C] (“CMS expects health spending to reach $5.6 trillion in 2025, with hospitals making up the largest share of spending ($1.8 trillion).").

275 The total U.S. gross domestic product is projected to be $30 trillion in 2025. *Gross Domestic Product, Second Quarter 2025 (Advance Estimate)*, Bureau Econ. Analysis (July 30, 2022), https://www.bea.gov/news/2022/gross-domestic-product-second-quarter-2022-advance-estimate [https://perma.cc/YY6H-XLYD].

276 Scott Hulver et al., *5 Key Facts About Medicaid and Hospitals*, Kaiser Fam. Found. (Mar. 5, 2025), https://www.kff.org/medicaid/5-key-facts-about-medicaid-and-hospitals/#:~:text=1.,of%20hospital%20discharges%20in%202023 [https://perma.cc/77PQ-V82W].

277 Kelly Gooch, *17 States Where Hospitals are the Largest Employers*, Becker’s Hosp. Rev. (May 12, 2020), https://www.beckershospitalreview.com/workforce/17-states-where-hospitals-are-largest-employers.html [https://perma.cc/5X99-6CZA]. Overall, health care is the largest employer in the United States. *See* Earlene K.P. Dowell, *Health Care Still Largest U.S. Employer*, U.S. Census Bureau, (Oct. 13, 2020), https://www.census.gov/library/stories/2020/10/health-care-still-largest-united-states-employer.html [https://perma.cc/8SER-X25R] ("According to the U.S. Census Bureau’s County Business Patterns (CBP), the 907,426 businesses in the Health Care and Social Assurance sector topped all others with 20 million employees over $1.0 trillion in annual payroll in 2018.”).

278 Gooch, *supra* note 277.

279
*Pennsylvania Hospitals’ Community Impact - Fiscal Year 2023*, Hosp. & Healthsystem Ass’n. Pa., https://www.haponline.org/About-PA-Hospitals/Community-Impact#:~:text=Hospitals%20and%20health%20systems%20in%20Pennsylvania%20have,nearly%20$1.9%20billion%20in%20federal%20research%20funding [https://perma.cc/69CW-7TVR] (last visited Aug. 26, 2025).

280
*Id.*

281 Imani Telesford et al., *What are the Recent Trends in Health Section Employment*? Peterson-KFF Health Sys. Tracker, (Mar. 27, 2024), https://www.healthsystemtracker.org/chart-collection/what-are-the-recent-trends-health-sector-employment/#Cumulative%20%25%20change%20in%20health%20sector%20employment%20by%20setting,%20February%202020%20-%20June%202023 [https://perma.cc/UQZ8-7J5Q] (“In April of 2020, health employment fell to 14.9 million from 16.2 million in 2019 (by -8.2%), while non-health employment fell by forteen percent%.”).

282
*See* Ayse Yilmaz, Obaid Zaman & Sushma Sharma, *Beyond Patient Care: Economic Impact of Pennsylvania Hospitals*, 31 Pa. Econ. Rev., Spring 2024, at 1, https://haponlinecontent.azureedge.net/resourcelibrary/fy2020-economic-analysis-white-paper-final.pdf [https://perma.cc/UUP3-CT64].

283 Holmes et al., *supra* note 249.

284 Holmes et al., *supra* note 249, at 478.

285 Holmes et al., *supra* note 249, at 480.

286 Holmes et al., *supra* note 249, at 481. The authors of this study suggested that rural hospitals faced particular financial pressure from Medicare’s switch to a prospective payment system of hospital reimbursement in the 1980s, noting the link between government reimbursement and hospital survival. *See id.*

287 Maria J. Perez-Villadoniga, Ana Rodriguez-Alvarez & David Roibas, *The Contribution of Resident Physicians to Hospital Productivity*, 23 Eur. J. Health Econ. 301, 301 (2021) (“In many countries, before becoming a physician, medical students must go through a long training process after completing their medical studies. Residency training generally takes place at a teaching hospital, where residents practice medicine under the supervision and instruction of fully licensed physicians. Teaching hospitals provide prospective future doctors with necessary education, which is a public good, insofar as well-trained physicians benefit society in general.”).

288
*See* Steven Ross Johnson, *Cost of Graduate Medical Education Stifling Ability to Bolster Physician Workforce*, Mod. Healthcare (May 4, 2019), https://www.modernhealthcare.com/providers/cost-graduate-medical-education-stifling-ability-bolster-physician-workforce/ [https://perma.cc/6D8J-T7AK] (discussing the high cost of training physicians and the limitations said cost places on hospitals’ ability to train).

289
*See* Marco A. Villagrana, Cong. Rsch. Serv., IF10960, Medicare Graduate Medical Education Payments: An Overview 1 (2022).

290
*Id.*

291
*State-by-State Graduate Medical Education Data*, Ass’n. Am. Med. Colls., https://web.archive.org/web/20240803192524/https://www.aamc.org/advocacy-policy/state-state-graduate-medical-education-data#expand (last visited Aug. 3, 2024) [hereinafter AAMC, *State GME Data*]. In 2018, Medicare supported 90,000 positions for medical residents. *Id. See also* Tim M. Henderson, Ass’n Am. Med. Colls., Medicaid Graduate Medical Education Payments: Results from the 2022 50-State Survey 2 (2023).

292 Henderson, *supra* note 291, at 4. Although many states make GME payments through Medicaid, almost one-fourth of state-based Medicaid GME funding was spent by one state, New York. Henderson, *supra* note 288, at 7.

293 Henderson, *supra* note 291, at 22.

294
*Id.* at 4.

295
*Id.* at 24.

296 AMA, 2023 Compendium of Graduate Medical Education Initiatives Report 4 (2023), https://www.ama-assn.org/system/files/2023-gme-compendium-report.pdf [https://perma.cc/M6JU-8L9D]. In 2012, total federal GME payments were $15 billion. Inst. Med. Nat’l Acads., Graduate Medical Education That Meets the Nation’s Health Needs 1 (Jill Eden et al. eds., 2014), https://www.aapmr.org/docs/default-source/advocacy/gme/institute-of-medicine---graduate-medical-education-report---meeting-the-nation-39-s-health-needs---july-2014.pdf [https://perma.cc/FC42-D9G4].

297 AAMC, *State GME Data*, *supra* note 291, at 4.

298 In 2013, Medicare paid $5.8 billion to hospitals for indirect medical education costs. *See* Report on Disproportionate Share Hospital Payments, *supra* note 108, at 10-12.

299
*See* Julie C. Spero et al., Cecil G. Sheps Ctr. for Health Servs. Rsch., GME in the United States: A Review of State Initiatives 25 (2013), https://www.shepscenter.unc.edu/wp-content/uploads/2013/09/GMEstateReview_Sept2013.pdf [https://perma.cc/XWV3-K9JR].

300
*See* Christopher L. Keough & Stephanie A. Webster, *Why Medicaid GME Funding Should Be Preserved*, 61 Healthcare Fin. Mgmt. 54, 54 (2007).

301
*See* John K. Iglehart, *Institute of Medicine Report on GME — A Call for Reform*, 372 New Eng. J. Med. 376, 378 (2015) (discussing the impact of cutting funding on teaching hospitals).

302
*See* Gabrielle Redford, *What Happens When a Teaching Hospital Closes?*, Ass’n Am. Med. Colls. (July 12, 2019), https://www.aamc.org/news-insights/what-happens-when-teaching-hospital-closes [https://perma.cc/8DUK-H6VK].

303
*Id.*

304
*See* Zirui Song, Vineet Chopra & Laurence F. McMahon, *Addressing the Primary Care Workforce Crisis*, 21 AM. J. Managed Care e452, e452 (2015). By administering the funding for GME, the federal and state governments can also assure that there is an ample supply of clinicians trained in primary care and essential specialties. *See id.*

305 U.S. Gov’t. Accountability Off., GAO-21-391, Physician Workforce: Caps on Medicare-Funded Graduate Medical Education at Teaching Hospitals (2021) (“Medicare sets caps on both of its types of physician graduate medical education (GME) payments (direct and indirect) to teaching hospitals.”).

306
*See* Lindsay McLaren et al., *Why Public Health Matters Today and Tomorrow: The Role of Applied Public Health Research*, 110 Can. J. Pub. Health 317, 317 (2019), https://www.ncbi.nlm.nih.gov/pmc/articles/PMC6964435/ [https://perma.cc/L4TG-HAX3] (“Public Health Is Critical to a Healthy, Fair, and Sustainable Society.”).

307 See *Top 10 Ways to Improve Health and Health Equity*, Ctr. for Am. Progress (Apr. 28, 2022), https://www.americanprogress.org/article/top-10-ways-to-improve-health-and-health-equity/ [https://perma.cc/4BRP-V9EJ] (listing “improve health care access and quality” as one of the 10 key priorities for improving the nation’s health).

308 Ctrs for Disease Control and Prevention, *Achievements in Public Health, 1900-1999: Control of Infectious Diseases*, 48 MMWR 621 (1999) (“Deaths from infectious diseases have declined markedly in the United States during the 20th century.”). Research has even shown an association between overall state spending on social assistance and adult survival from cancer. *See* Justin M. Barnes et al., *State Public Assistance Spending and Survival Among Adults with Cancer*, 6 JAMA Network Open 1, 1 (2013).

309 Vaccination has been called “one of the most powerful means to save lives and to increase the level of health of mankind.” Paolo Bonanni, *Demographic Impact of Vaccination: A Review*, 17 Vaccine S120, S120 (1999).

310
*Vaccines and Immunization*, World Health Org., https://www.who.int/health-topics/vaccines-and-immunization#tab=tab_1 [https://perma.cc/D6T4-V5NL] (last visited Aug. 26, 2025).

311
*See U.S. Vaccine Names*, Ctrs. for Disease Control & Prevention (June 23, 2025), https://www.cdc.gov/vaccines/hcp/vaccines-us/?CDC_AAref_Val=https://www.cdc.gov/vaccines/vpd/vaccines-list.html [https://perma.cc/P3ZH-ZF99].

312
*See State Vaccination Requirements*, Ctrs. for Disease Control & Prevention (Aug. 6, 2024), https://www.cdc.gov/vaccines/php/requirements-laws/state-vaccination-requirements.html?CDC_AAref_Val= [https://perma.cc/C3NJ-DEE6]. However, in 2025, Florida announced plan to repeal all vaccines mandates in the state. Judy Stone, *Florida Plans To Eliminate All Vaccine Mandates — What That Means for Us.*, Forbes (Sep. 4, 2025), https://www.forbes.com/sites/judystone/2025/09/04/florida-plans-to-eliminate-all-vaccine-mandates-what-that-means-for-us/ [https://perma.cc/R8KX-QNH4].

313
*See* Charlene M.C. Rodrigues & Stanley A. Plotkin, *Impact of Vaccines; Health, Economic and Social Perspectives*, 11 Frontiers in Microbiology, no. 1526, July 2020, at 4-5 (“Where a sufficiently high portion of the population are vaccinated, transmission of the infecting agent is halted thereby protecting the unvaccinated, who may be those too young, too vulnerable, or too immunosuppressed to receive vaccines.”).

314
*The Relationship Between Vaccines and Herd Immunity*, Colum. Mailman Sch. of Pub. Health (Apr. 9, 2021), https://www.publichealth.columbia.edu/public-health-now/news/relationship-between-vaccines-and-herd-immunity [https://perma.cc/W4RL-DNNV].

315
*See, e.g.*, *id.* (citing estimates to achieve herd immunity for measles (95%), polio (80%), and COVID-19 (70-90%)).

316 Tae Hyong Kim et al., *Vaccine Herd Effect*, 43 Scandinavian J. Infectious Diseases 683, 683 (“The herd effect or herd immunity is an attractive way to extend vaccine benefits beyond the directly targeted population.”).

317
*See* Folland et al., *supra* note 142, at 532-33 (modeling limitations on influenza vaccinations due to cost).

318
*See id.* From the perspective of economic efficiency, the broader marginal benefit from the vaccine is greater because of the externality. Since individuals are typically concerned only with the benefit to themselves, the price will not reflect this greater benefit. As a result, it may be underpriced and therefore underproduced. *See id.*

319 Thomas L. Schlnker et al., *Measles Herd Immunity: The Association of Attack Rates with Immunization Rates in Preschool Children*, 267 [J]AMA 823, 826 (1992) (“An important finding of this study was the highly significant correlation between increasing immunization coverage and decreasing measles transmission. In particular, our analysis suggests a threshold of immunization coverage above which widespread measles transmission does not appear to occur.”); *see also* ANDRULIS, *supra* note 92, at 132.

320
*See* Bryan L. Boulier, Tejwant S. Datta & Robert S. Goldfarb, *Vaccination Externalities*, 7 B.E. J. Econ. Analysis & Pol’y, no. 23, 2007, https://www.degruyter.com/document/doi/10.2202/1935-1682.1487/html [https://perma.cc/S2FZ-4D5E] (“Vaccination provides indirect benefits to the unvaccinated … . The size of the externality is not necessarily monotonic in the number of vaccinated, vaccine efficacy, nor disease infectiousness. Moreover, its magnitude can be remarkably large. In particular, the marginal externality of a vaccination can be greater than one case of illness prevented among the nonvaccinated, so its omission from policy analyses implies serious biases.”).

321 Hugh Waters & Marlon Graf, *Chronic Diseases are Taxing our Health Care System and Our Economy.* Stat (May 31, 2018), https://www.statnews.com/2018/05/31/chronic-diseases-taxing-health-care-economy/ [https://perma.cc/C3GU-SMU7].

322 T. Benfield et al., *Influence of Diabetes and Hyperglycaemia on Infectious Disease Hospitalisation and Outcome*, 50 Diabetologia 549, 549 (2007).

323
*See Fast Facts: Health and Economic Costs of Chronic Diseases*, Ctrs. for Disease Control & Prevention (July 12, 2024), https://www.cdc.gov/chronic-disease/data-research/facts-stats/?CDC_AAref_Val=https://www.cdc.gov/chronicdisease/about/costs/index.htm [https://perma.cc/T46C-FNKW]; *Chronic Disease Prevention in the U.S. - Statistics & Facts*, Statista (Nov. 6, 2024), https://www.statista.com/topics/8951/chronic-disease-prevention-in-the-us/#topicOverview [https://perma.cc/UJX9-VQU5] (“The CDC estimates that six in ten adults in the United States currently live with a chronic disease such as cancer, heart disease, or diabetes. Chronic diseases are among the leading causes of death in the United States with heart disease and cancer alone accounting for around forty percent of all deaths.”).

324 U.S. Census Bureau, Statistical Abstract of the United States 874 (1999), https://www2.census.gov/library/publications/1999/compendia/statab/119ed/tables/sec31.pdf [https://perma.cc/3VWZ-SE4J].

325
*See* Jason D. Buxbaum et al., *Contributions of Public Health, Pharmaceuticals, and Other Medical Care to US Life Expectancy Changes, 1990-2015*, 39 Health Affs. 1546, 1546 (2020) (stating that forty-four percent of improved life expectancy was attributable to public health).

326
*See id*; Mohammad Rahman et al., *Determinants of Life Expectancy in Most Polluted Countries: Exploring the Effect of Environmental Degradation*, PlosOne Jan. 21, 2022, at 1, 4.

327
*See* Felix Stein & Devi Sridhar, *Health as a “Global Public Good”: Creating a Market for Pandemic Risk*, BMJ Online, 2017, at 3, https://www.bmj.com/content/bmj/358/bmj.j3397.full.pdf [https://perma.cc/5KRS-JQ53].

328
*See* Suerie Moon, John-Arne Rottingen & Julio Frenk, *Global Public Goods for Health: Weaknesses and Opportunities in the Global Health System*, 12 Health Econ., Pol’y, & L. 195, 195 (2017), https://pubmed.ncbi.nlm.nih.gov/28332461/ [https://perma.cc/88FV-XHKQ].

329 Families First Coronavirus Response Act (FFCRA), Pub. L. No. 116-127, §§ 1702, 6008, 134 Stat. 178, 183, 208-09 (2020) (describing state and local government duty to provide data to the CDC and discussing the requirement that recipients utilize funds to cover vaccines and treatment related to COVID-19). Enhancing and stabilizing Medicaid were key components of the federal government’s response to the COVID pandemic. *Id.* The Coronavirus Aid, Response and Economic Security CARES) Act increased the federal share of Medicaid funding by 6.2 percent during the time that the COVID national public health emergency (PHE) was in effect. *See* Sara Rosenbaum , Morgan Handley & Rebecca Morris , *Winding Down Continuous Enrollment for Medicaid Beneficiaries When the Public Health Emergency Ends*, The Commonwealth Fund (Jan. 7, 2021), 10.26099/bw1x-3r88. It also imposed a moratorium on eligibility reviews and disenrollment by states during the PHE. *See id.*

330
*See* Ctr. for the Developing Child, *Lifelong Health and Well-being*, Harv. Univ., https://developingchild.harvard.edu/science/deep-dives/lifelong-health/ [https://perma.cc/8QH9-VENA] (last visited Jan. 23, 2024).

331 Rosemarie B. Hakim et al., *Medicaid and the Health of Children*, 22 Health Care Fin. Rev. 133, 133 (2000) (“Since its inception in the 1960s, the Medicaid Program has provided health insurance coverage to low-income children and their families.”); *see also*, Judith D. Moore & David G. Smith, *Legislating Medicaid: Considering Medicaid and Its Origins*, 27 Health Care Fin. Rev. 45, 47-48 (2005) (noting a statement by Wilbur Mills, one of Medicaid’s key Congressional sponsors, that “a Medicare Hospital insurance program for the aged alone was not sufficient to meet the many medical needs of the aged, blind, and disabled or the mothers and children receiving aid for dependent children”).

332 42 U.S.C. §§ 1396a(a)(43), 1396d(a)(4)(B); *see also* 42 C.F.R. § 441.50 (2024); Jane Perkins & Sarah Somers. *Medicaid’s Gold Standard Coverage for Children and Youth: Past, Present, and Future.* 30 Annals Health L. & Life Scis. 153, 158 (2021).

333

Anne Rossier Marcus et al., *Medicaid Covered Births, 2008 Through 2010, in the Context of the Implementation of Health Reform*, 23 Women’s Health Issues e273, e273 (2013), 10.1016/j.whi.2013.06.006 23993475.

334
*Access for Children Covered by Medicaid and CHIP*, MACPAC (July 16, 2023), https://www.macpac.gov/subtopic/access-for-children-covered-by-medicaid-and-chip/ [https://perma.cc/GX6L-ESZ8].

335 Julia Paradise, *The Impact of the Children’s Health Insurance Program (CHIP): What Does the Research Tell Us?*, Kaiser Fam. Found. (July 17, 2014), https://www.kff.org/report-section/the-impact-of-the-childrens-health-insurance-program-chip-issue-brief/ [https://perma.cc/HDG7-4G6P].

336 Hakim et al., *supra* note 331, at 136-37.

337 Hakim et al., *supra* note 331, at 137.

338 Hakim et al., *supra* note 331, at 136.

339
*See* Michel H. Bourdreaux et al., *The Long-Term Impacts of Medicaid Exposure in Early Childhood: Evidence from the Program’s Origin*, 45 J. Health Econ. 161, 161 (2016).

340
*See* Sarah R. Cohodes et al., *The Effect of Child Health Insurance Access on Schooling: Evidence from Public Insurance Expansions*, 51 J. Hum. Res. 727, 727 (2016).

341
*See* David W. Brown et al., *Medicaid as an Investment in Children: What is the Long-Term Impact on Tax Receipts?*, (Nat’l Bureau of Econ. Rsch., Working Paper No. 20835, 2015), https://www.nber.org/system/files/working_papers/w20835/w20835.pdf [https://perma.cc/76MM-GHJG].

342
*Id.*

343
*See* MaryBeth Musumeci, Molly O’Malley Watts & Priya Chidambaram, *Key State Policy Choices About Medicaid Home and Community-Based Services*, Kaiser Fam. Found. (Feb. 4, 2020), https://www.kff.org/medicaid/issue-brief/key-state-policy-choices-about-medicaid-home-and-community-based-services/ [https://perma.cc/DX6P-J4Q2].

344
*See* Douglas L. Leslie et al., *The Effects of Medicaid Home and Community-Based Services Waivers on Unmet Needs Among Children with Autism Spectrum Disorder*, 55 Med. Care 57, 57 (2017). As discussed in section II, these waivers have also been found to increase the likelihood that parents of disabled children will be able continue working, thereby easing financial stresses on many families. *See* Kiley J. McLean et al., *United States Medicaid Home and Community-Based Services for People with Intellectual and Developmental Disabilities: A Scoping Review*, 34 J. Applied Rsch. Intell. Disabilities 684, 691 (2020).

345
*See* Michelle LaClair et al., *The Effect of Medicaid Waivers on Ameliorating Racial/Ethnic Disparities Among Children with Autism*, 54 Health Serv. Rsch. 912, 912 (2019).

346
*Direct Economic Contributions*, Nat’l Inst. of Health (Apr. 18, 2025), https://www.nih.gov/about-nih/what-we-do/impact-nih-research/serving-society/direct-economic-contributions [https://perma.cc/6CGP-FAQF] (“With an annual budget of more than $45 billion, NIH is the largest single public funder of biomedical and behavioral research in the world.”).

347
*See* Daniel Kevles , *Medicare, Medicaid, and Pharmaceuticals: The Price of Innovation*, Health Affs.: Forefront (Nov. 20, 2014), 10.1377/hblog20141120.042872 25876383. All Medicaid programs cover prescription drugs and thereby finance an important market for these innovations. *See Medicaid Prescription Drug Laws and Strategies*, Nat’l Conf. State Legislatures (Aug. 27, 2021), https://www.ncsl.org/health/medicaid-prescription-drug-laws-and-strategies [https://perma.cc/SPF4-SXHD].

348 Nat’l Acad. Soc. Ins., Medicare and the American Social Contract - Restructuring Medicare for the Long Term Project: Final Report of the Study Panel on Medicare’s Larger Social Role 24 (1999), https://web.archive.org/web/20160315173424/https://www.nasi.org/usr_doc/med_report_soc_contract.pdf [https://perma.cc/N8CX-CUZ5].

349
*See* Rachel Dolan & Marina Tian, *Pricing and Payment for Medicaid Prescription Drugs*, Kaiser Fam. Found. (Jan. 23, 2020), https://www.kff.org/medicaid/issue-brief/pricing-and-payment-for-medicaid-prescription-drugs/ [https://perma.cc/JH8T-DN9A].

350
*See* Cong. Budget Off., Prescription Drugs: Spending, Use, and Prices 8 (2022), https://www.cbo.gov/system/files/2022-01/57050-Rx-Spending.pdf [https://perma.cc/VY47-P2KB]; *see also* Juliette Cubanski et al., *How Does Prescription Drug Spending and Use Compare Across Large Employer Plans, Medicare Part D, and Medicaid?*, Kaiser Fam. Found. (May 20, 2019), https://www.kff.org/medicare/issue-brief/how-does-prescription-drug-spending-and-use-compare-across-large-employer-plans-medicare-part-d-and-medicaid/ [https://perma.cc/QN7G-EPQZ] (reporting that ten percent of total U.S. Retail Prescription Drug Spending came from Medicaid in 2017). Some industry critics contend that the value of the Medicaid market to drug companies is too large. *See* Rachel E. Sachs et al., *Confronting State Medicaid Drug Spending Pressures*, 324 JAMA 1831, 1831-32 (2020) ("Across the country, state Medicaid budgets are under increasing strain, driven by factors including the high and increasing prices of prescription drugs.”).

351 Cong. Budget Off., *supra* note 350, at 8.

352
*May 2025 Medicaid & CHIP Enrollment Data Highlights*, *supra* note 89. Medicaid and CHIP covered more than 78 million people in 2025. *Id.*

353 David Himmelstein et al., *Medical Bankruptcy: Still Common Despite the Affordable Care Act*, 198 Am. J. Pub. Health 431, 431 (2019).

354 Aubrey Whelan, *At Northeast Philly’s Only City-Run Primary Care Clinic, Patients Wait up to a Year to Get an Appointment*, Phila. Inquirer (May 26, 2023), https://www.inquirer.com/health/primary-care-northeast-philadelphia-clinic-wait-times-20230526.html [https://perma.cc/XMB8-P7Y2]; *See What is a Health Center?*, Health Res. & Servs. Admin. (Apr. 2025), https://bphc.hrsa.gov/about-health-centers/what-health-center [https://perma.cc/83PK-QNFN]. Long wait times are common at outpatient clinics around the world. *See* Mohammadkarim Bahadori et al., *Factors Affecting the Overcrowding in Outpatient Healthcare*, 6 J. Educ. & Health Promotion, Apr. 2017, at 1.

355
*See* Andrew P. Wilper et al., *A National Study of Chronic Disease Prevalence and Access to Care in Uninsured U.S. Adults*, 149 Annals Internal Med. 170, 173-74 (2008).

356
*See id.*

357
*See id.* at 170.

358

Wilhelmine Miller et al., *Covering the Uninsured: What is It Worth?*, Health Affs. (Dec. 5, 2018), 10.1377/hlthaff.W4.157 15451984.

359 Inst. of Med., Unequal Treatment: Confronting Racial and Ethnic Disparities in Health Care 1 (Brian D. Smedley et al. eds., 2003). Additionally, several studies showed significant racial disparities in receiving cancer diagnostic tests and higher morbidity among Black patients diagnosed with cancer even when testing detected illness earlier. *Id.* at 5. Racial disparities were also seen in diabetes care, renal disease, pediatric care, maternal and child health, mental health, rehabilitative and nursing home services, and many surgical procedures. *Id.* at 6.

360 Miller et al., *supra* note 358, at 157.

361
*Id.*

362
*See* Jeffrey D. Colvin et al., *Financial Loss for Inpatient Care of Medicaid-Insured Children*, 170 JAMA Pediatrics 1055, 1056 (2016) (stating that “the care of patients with Medicaid coverage also contributes to uncompensated costs because Medicaid typically reimburses below hospital costs. In 2014, hospital financial losses from Medicaid underpayment totaled $14.1 billion”).

363
*See* Gary J. Young et al., *Impact of ACA Medicaid Expansion on Hospitals’ Financial Status*, 64 J. Healthcare Mgmt. 91, 91 (2019).

364
*Provider Payment and Delivery Systems*, MACPAC, https://www.macpac.gov/medicaid-101/provider-payment-and-delivery-systems/#:~:text=Medicaid%20FFS%20payment%20rates%20for%20physician%20services,care%20(Decker%202012%2C%20Cunningham%20and%20May%202006).&text=On%20average%2C%20Medicaid%20FFS%20physician%20payment%20rates,this%20varies%20greatly%20by%20state%20and%20service [https://perma.cc/WF5Q-ZYHH] (last visited Aug. 26, 2025).

365
*See* James M. Perrin et al., *Medicaid and Child Health Equity*, 383 New Eng. J. Med. 2595, 2597 (2020); *see also*, Daniel Polsky et al., *Appointment Availability After Increases in Medicaid Payments for Primary Care*, 372 New Eng. J. Med. 537, 538 (2015) (“Lower payments have been cited as a critical barrier to access for primary care among Medicaid enrollees and are associated with lower provider availability for Medicaid patients.”) (footnote omitted).

366
*See* Abe Dunn et al., *A Denial a Day Keeps the Doctor Away* (Nat’l Bureau Econ. Rsch., Working Paper No. 29010, 2023), https://www.nber.org/papers/w29010 [https://perma.cc/69C8-U2G3].

367
*See* Perrin et al., *supra* note 365, at 2597.

368
*See* Polsky et al., *supra* note 365, at 538. (“Provider access is of particular concern for the Medicaid program, which is set to absorb the bulk of newly insured persons in many states [under the ACA], because Medicaid typically reimburses providers at much lower payment rates than those of Medicare and commercial insurers for the same services.”).

369
*See* Gideon Lukens, Ctr. on Budget & Pol’y Priorities, Medicaid Coverage Gap Affects Even Larger Group Over Time Than Estimates Indicate (Sep. 3, 2021) (discussing that loss of Medicaid coverage due to income volatility affected Black and Latino households the most).

370 Jim P. Stimpson & Fernando A. Wilson, *Medicaid Expansion Improved Health Insurance Coverage for Immigrants, But Disparities Persist*, 37 Health Affs. 1656, 1656 (2018).

371
*See* Joel S. Weissman et al., *Limits to the Safety Net: Teaching Hospital Faculty Report on Their Patients’ Access to Care*, 22 Health Affs. 156, 156 (2003) (reporting the observation of medical school faculty that initial treatment of an uninsured patient at a teaching hospital does not guarantee access to specialty and other referral services).

372 Ruohua Annetta Zhou et al., *The Uninsured Do Not Use the Emergency Department More — They Use Other Care Less*, 36 Health Affs. 2115, 2120 (2017) (reporting results of study that found that those without insurance use nonemergency hospital care less than those with insurance and explaining as a reason that the uninsured “may be legally denied care in non-ED settings”).

373 Michelle N. Diamond, *Legal Triage for Healthcare Reform: The Conflict Between the ACA and EMTALA*, 43 Colum. Human Rts. L. Rev. 255, 262 (2011) (“In short, EMTALA requires hospitals to provide emergency medical care to all individuals — including undocumented immigrants — regardless of their ability to pay for treatment, but does not require or reimburse non-emergency medical care.”).

374
*See* Noam Levey, *Investigation: Many U.S. Hospitals Sue Patients for Debts or Threaten Their Credit*, NPR (Dec. 21, 2022, 05:01 ET), https://www.npr.org/sections/health-shots/2022/12/21/1144491711/investigation-many-u-s-hospitals-sue-patients-for-debts-or-threaten-their-credit [https://perma.cc/L4KU-NEQE].

375
*See* Michael Sainato, *‘I Live on the Street Now’: How Americans Fall into Medical Bankruptcy*, The Guardian (Nov. 14, 2019, 02:00 ET), https://www.theguardian.com/us-news/2019/nov/14/health-insurance-medical-bankruptcy-debt [https://perma.cc/2PZF-DEQC].

376
*See* Sarah Kliff & Jessica Silver-Greenberg, *Nonprofit Health System Pauses Policy of Cutting Off Care for Patients in Debt*, N.Y. Times (June 9, 2023), https://www.nytimes.com/2023/06/09/health/allina-health-nonprofit-medical-debt.html [https://perma.cc/WBM9-AJ4Q].

377 Junaid Nabi, et al., *Access Denied: The Relationship Between Patient Insurance Status and Access to High-Volume Hospitals.* 127 Cancer 577, 582-84 (2021).

378
*See* Robert I. Field et al., *Private Equity in Health Care: Barbarians at the Gate?*, 15 Drexel L. Rev. 821, 821-22 (2023) (describing acquisitions of long-term care facilities by private equity firms).

379
*See* Hum. Rts. Watch, US: Concerns of Neglect in Nursing Homes 5-7, 18 (2021), available at https://www.hrw.org/sites/default/files/media_2022/02/drd_nursinghome0521_brochure_PRINT_0.pdf [https://perma.cc/V6YC-ZYHG].

380 Field et al., *supra* note 378, at 890 (discussing that health care entities purchased by private equity firms are often non-compliant with billing and other regulations, costing the government and taxpayers money).

381 Robert Gebeloff, Kaie Thomas & Jessica Silver-Greenberg, *How Nursing Homes’ Worst Offenses Are Often Hidden from the Public*, N.Y. Times (Dec. 10, 2021), https://www.nytimes.com/2021/12/09/business/nursing-home-abuse-inspection.html [https://perma.cc/5TGD-UGHX].

382
*Id.*

383
*See* George Gao, *How Do Americans Stand Out from the Rest of the World?*, Pew Rsch. Ctr. (Mar. 12, 2015), https://www.pewresearch.org/short-reads/2015/03/12/how-do-americans-stand-out-from-the-rest-of-the-world/ [https://perma.cc/T68B-PC6P] (discussing Americans’ emphasis on individualism and work ethic); *see also* Patrick Carroll & Dan Sanchez, *Individualism: A Deeply American Philosophy*, Found. for Econ. Educ. (July 2, 2022), https://fee.org/resources/individualism-a-deeply-american-philosophy/ [https://perma.cc/7YKM-Q2M9] (“Individualism is a philosophy that views people first and foremost as unique individuals rather than as members of a group. It emphasizes the importance of independence, individuality, and autonomy.”).

384 Melissa Mohr, *How the ‘Bootstrap’ Idiom Became a Cultural Ideal. Christian Science Monitor*, Christian Sci. Monitor (Oct. 4, 2021), https://www.csmonitor.com/The-Culture/In-a-Word/2021/1004/How-the-bootstrap-idiom-became-a-cultural-ideal [https://perma.cc/GHL4-K4CG].

385
*See* Rosenbaum, *supra* note 27 (discussing that work requirements are put in place based on a belief that health care should be limited to those who “deserve[]” it).

386
*See* Nicole Huberfeld & Jessica L. Roberts, *Health Care and the Myth of Self-Reliance*, 57 B.C. L. Rev. 1, 23 (2016) (“The concept of self-reliance, as a principle in modern American social and political discourse, is a myth.”).

387
*See* Field, *supra* note 378 at 10-12 (describing how every major sector of the health care industry, including pharmaceuticals, hospitals, the medical profession, and insurance, has relied on government funding throughout its history). The central importance of government funding and support can also be seen across major industries, including technology, automobiles, telecommunications, and homebuilding. *Id.*

388 Some of these proposals are described in note 22 *supra.* They include proposals, such as work requirements, that were enacted by Congress in the 2025 budget bill. *See supra* note 3 and accompanying text.

389 Elizabeth Williams et al. *Implications of Continuous Eligibility Policies for Children’s Medicaid Enrollment Churn*, Kaiser Fam. Found. (Dec. 21, 2022), https://www.kff.org/medicaid/issue-brief/implications-of-continuous-eligibility-policies-for-childrens-medicaid-enrollment-churn/ [https://perma.cc/7GU7-NEXR].

390 Frederic Blavin et al., *The Effects of Express Lane Eligibility on Medicaid and CHIP Enrollment Among Children*, 49 Health Serv. Rsch. 1268, 1268 (2014).

391 See Robert I. Field, *The Devil in the Details: State Medicaid Administrative Rules as Enrollment Policy*, 23 Hou. J. Health L. & Pol’y 11, 32, 41 (2024).

